# Supplementary material for: Estimation of environment stability for fruit yield and capsaicin content by using two models in Capsicum chinense Jacq. (Ghost Pepper) with multi-year evaluation
Source: PeerJ. 2024 Jul 10;12:e17511. doi: 10.7717/peerj.17511 (PMC11246027; doi:10.7717/peerj.17511)
Supplement: Supplemental Information 1 [file peerj-12-17511-s001.docx]

Supplementary Material

**Estimation of environment stability for fruit yield and capsaicin content by using two models in *Capsicum chinense* Jacq. (Ghost Pepper)** **with multi-year evaluation**

Joyashree Baruah^1,2^, Twahira Begum^2^, Sanjoy Kumar Chanda^2^ and Mohan Lal^2^*

^1^Department of Botany, Eastern Karbi Anglong College, Sarihajan, Assam-782480, India

^2^Agrotechnology and Rural Development Division, CSIR-North East Institute of Science and Technology (NEIST), Jorhat, Assam-785006, India

## Supplementary Tables

Table S1: Pedigree number and capsaicin content (%) of 120 genotypes of *Capsicum chinense* Jacq. used in the study.

| Line. No | Pedigree | Capsaicin content (%) | Line. No | Pedigree | Capsaicin content (%) |
| --- | --- | --- | --- | --- | --- |
| 1 | RRLJ-BJ-1 | 2.073 | 61 | RRLJ-BJ-61 | 2.096 |
| 2 | RRLJ-BJ-2 | 1.827 | 62 | RRLJ-BJ-62 | 1.881 |
| 3 | RRLJ-BJ-3 | 1.787 | 63 | RRLJ-BJ-63 | 2.284 |
| 4 | RRLJ-BJ-4 | 1.915 | 64 | RRLJ-BJ-64 | 2.063 |
| 5 | RRLJ-BJ-5 | 2.965 | 65 | RRLJ-BJ-132 | 2.407 |
| 6 | RRLJ-BJ-6 | 1.748 | 66 | RRLJ-BJ-66 | 2.541 |
| 7 | RRLJ-BJ-7 | 1.611 | 67 | RRLJ-BJ-67 | 1.710 |
| 8 | RRLJ-BJ-8 | 1.597 | 68 | RRLJ-BJ-68 | 2.597 |
| 9 | RRLJ-BJ-9 | 1.961 | 69 | RRLJ-BJ-142 | 1.600 |
| 10 | RRLJ-BJ-10 | 1.915 | 70 | RRLJ-BJ-70 | 3.222 |
| 11 | RRLJ-BJ-11 | 2.004 | 71 | RRLJ-BJ-71 | 1.729 |
| 12 | RRLJ-BJ-12 | 1.601 | 72 | RRLJ-BJ-72 | 2.796 |
| 13 | RRLJ-BJ-13 | 2.236 | 73 | RRLJ-BJ-73 | 1.904 |
| 14 | RRLJ-BJ-14 | 1.495 | 74 | RRLJ-BJ-74 | 1.977 |
| 15 | RRLJ-BJ-15 | 1.473 | 75 | RRLJ-BJ-75 | 2.093 |
| 16 | RRLJ-BJ-16 | 1.327 | 76 | RRLJ-BJ-76 | 2.028 |
| 17 | RRLJ-BJ-17 | 2.806 | 77 | RRLJ-BJ-77 | 1.608 |
| 18 | RRLJ-BJ-18 | 3.628 | 78 | RRLJ-BJ-78 | 1.555 |
| 19 | RRLJ-BJ-19 | 2.576 | 79 | RRLJ-BJ-79 | 1.501 |
| 20 | RRLJ-BJ-135 | 1.373 | 80 | RRLJ-BJ-80 | 1.518 |
| 21 | RRLJ-BJ-21 | 2.197 | 81 | RRLJ-BJ-81 | 1.962 |
| 22 | RRLJ-BJ-22 | 2.415 | 82 | RRLJ-BJ-82 | 2.239 |
| 23 | RRLJ-BJ-136 | 1.989 | 83 | RRLJ-BJ-83 | 1.686 |
| 24 | RRLJ-BJ-24 | 2.641 | 84 | RRLJ-BJ-84 | 2.078 |
| 25 | RRLJ-BJ-25 | 1.460 | 85 | RRLJ-BJ-85 | 2.187 |
| 26 | RRLJ-BJ-26 | 2.270 | 86 | RRLJ-BJ-86 | 1.835 |
| 27 | RRLJ-BJ-27 | 2.044 | 87 | RRLJ-BJ-87 | 2.085 |
| 28 | RRLJ-BJ-28 | 1.715 | 88 | RRLJ-BJ-88 | 2.759 |
| 29 | RRLJ-BJ-29 | 2.008 | 89 | RRLJ-BJ-89 | 2.375 |
| 30 | RRLJ-BJ-30 | 1.484 | 90 | RRLJ-BJ-90 | 2.515 |
| 31 | RRLJ-BJ-31 | 1.674 | 91 | RRLJ-BJ-91 | 1.891 |
| 32 | RRLJ-BJ-32 | 2.066 | 92 | RRLJ-BJ-92 | 1.364 |
| 33 | RRLJ-BJ-33 | 1.685 | 93 | RRLJ-BJ-93 | 1.365 |
| 34 | RRLJ-BJ-34 | 2.069 | 94 | RRLJ-BJ-94 | 1.455 |
| 35 | RRLJ-BJ-137 | 1.603 | 95 | RRLJ-BJ-95 | 1.915 |
| 36 | RRLJ-BJ-36 | 2.219 | 96 | RRLJ-BJ-96 | 1.667 |
| 37 | RRLJ-BJ-138 | 2.395 | 97 | RRLJ-BJ-97 | 2.054 |
| 38 | RRLJ-BJ-38 | 1.848 | 98 | RRLJ-BJ-98 | 3.223 |
| 39 | RRLJ-BJ-39 | 2.253 | 99 | RRLJ-BJ-99 | 2.401 |
| 40 | RRLJ-BJ-40 | 2.227 | 100 | RRLJ-BJ-100 | 2.695 |
| 41 | RRLJ-BJ-41 | 1.969 | 101 | RRLJ-BJ-101 | 2.402 |
| 42 | RRLJ-BJ-42 | 2.956 | 102 | RRLJ-BJ-107 | 4.188 |
| 43 | RRLJ-BJ-43 | 2.125 | 103 | RRLJ-BJ-108 | 1.850 |
| 44 | RRLJ-BJ-44 | 1.776 | 104 | RRLJ-BJ-109 | 2.230 |
| 45 | RRLJ-BJ-45 | 2.639 | 105 | RRLJ-BJ-110 | 1.638 |
| 46 | RRLJ-BJ-139 | 1.995 | 106 | RRLJ-BJ-111 | 3.125 |
| 47 | RRLJ-BJ-47 | 2.049 | 107 | RRLJ-BJ-112 | 2.945 |
| 48 | RRLJ-BJ-48 | 2.011 | 108 | RRLJ-BJ-113 | 2.193 |
| 49 | RRLJ-BJ-49 | 1.534 | 109 | RRLJ-BJ-126 | 2.326 |
| 50 | RRLJ-BJ-50 | 1.952 | 110 | RRLJ-BJ-116 | 2.195 |
| 51 | RRLJ-BJ-140 | 1.476 | 111 | RRLJ-BJ-127 | 2.467 |
| 52 | RRLJ-BJ-141 | 1.705 | 112 | RRLJ-BJ-119 | 1.771 |
| 53 | RRLJ-BJ-53 | 2.186 | 113 | RRLJ-BJ-120 | 1.494 |
| 54 | RRLJ-BJ-54 | 2.472 | 114 | RRLJ-BJ-121 | 2.120 |
| 55 | RRLJ-BJ-55 | 1.942 | 115 | RRLJ-BJ-122 | 1.400 |
| 56 | RRLJ-BJ-56 | 1.433 | 116 | RRLJ-BJ-123 | 2.274 |
| 57 | RRLJ-BJ-57 | 2.123 | 117 | RRLJ-BJ-125 | 1.842 |
| 58 | RRLJ-BJ-58 | 2.260 | 118 | RRLJ-BJ-129 | 2.522 |
| 59 | RRLJ-BJ-59 | 2.305 | 119 | RRLJ-BJ-130 | 2.027 |
| 60 | RRLJ-BJ-60 | 3.979 | 120 | RRLJ-BJ-131 | 2.535 |

Supplemental Table S2(A): Estimation of mean and stability parameters for capsaicin content, fruit length and girth, yield per plant, number of fruits per plant and days to maturity.

|  | Capsaicin content (%) | | | Fruit length (cm) | | | Fruit girth (cm) | | | Fruit yield/plant (g) | | | No. of fruits/plant | | | Days to maturity | | |
| --- | --- | --- | --- | --- | --- | --- | --- | --- | --- | --- | --- | --- | --- | --- | --- | --- | --- | --- |
| Line No. | Mean (µ) | βi | σ²di | Mean (µ) | βi | σ²di | Mean (µ) | βi | σ²di | Mean (µ) | βi | σ²di | Mean (µ) | βi | σ²di | Mean (µ) | βi | σ²di |
| 1 | 2.07 | 0.49 | -0.01 | 6.700 | 1.53 | -0.02 | 7.456 | 1.33 | 0.00 | 422.17 | 1.20 | 693.89 | 59.11 | 0.95 | 3.30 | 178.00 | 0.62 | 0.22 |
| 2 | 1.83 | 0.58 | 0.02 | 6.900 | 0.83 | 0.25 | 7.333 | 0.95 | 0.78 | 432.52 | 0.96 | 3707.86 | 60.33 | 0.82 | 1.30 | 175.56 | 0.63 | 1.97 |
| 3 | 1.79 | 1.06 | -0.01 | 6.722 | 0.49 | 0.00 | 7.356 | 0.60 | 0.02 | 325.23 | 0.40 | 239.27 | 46.11 | 0.36 | 1.39 | 173.56 | 0.62 | -0.51 |
| 4 | 1.92 | 0.27 | -0.01 | 6.700 | 1.05 | 0.63 | 7.322 | 1.02 | 0.15 | 409.97 | -0.29 | 1340.2 | 69.11 | 1.12 | 8.19 | 172.22 | 0.80 | -0.50 |
| 5 | 2.96 | 2.15 | 0.01 | 7.467 | 0.54 | 0.03 | 7.889 | 0.87 | 0.13 | 595.15 | 1.34 | -88.29 | 77.22 | 1.33 | -10.40 | 175.22 | 1.22 | 3.45 |
| 6 | 1.75 | 0.72 | 0.00 | 6.767 | 1.05 | 0.04 | 7.222 | 1.11 | 0.07 | 355.86 | 0.86 | 1369.29 | 50.56 | 0.76 | 9.34 | 176.11 | 0.63 | -0.17 |
| 7 | 1.61 | 0.95 | 0.03 | 6.600 | 1.25 | -0.02 | 7.256 | 1.33 | 0.00 | 305.69 | 0.98 | 6144.98 | 43.78 | 0.93 | 103.62 | 176.56 | 0.56 | -0.48 |
| 8 | 1.60 | 1.71 | -0.01 | 7.800 | 1.47 | -0.01 | 7.244 | 1.30 | 0.00 | 446.61 | 1.13 | 65.12 | 59.11 | 0.87 | 17.31 | 173.78 | 0.57 | 0.34 |
| 9 | 1.96 | 0.24 | -0.01 | 6.978 | 2.17 | -0.02 | 7.511 | 1.98 | 0.01 | 421.76 | 1.38 | 4605.81 | 57.89 | 0.90 | 65.79 | 169.33 | 0.42 | -0.51 |
| 10 | 1.92 | 0.12 | -0.01 | 7.178 | 0.76 | -0.03 | 7.789 | 0.87 | -0.03 | 394.22 | 0.76 | 983.88 | 52.56 | 0.70 | 16.59 | 173.67 | 1.32 | 2.92 |
| 11 | 2.00 | 0.23 | -0.01 | 6.511 | 1.15 | 0.06 | 6.844 | 1.19 | 0.02 | 269.59 | 0.81 | -438.51 | 40.11 | 0.80 | -8.25 | 168.67 | 0.39 | -0.45 |
| 12 | 1.60 | 0.21 | -0.01 | 6.744 | 1.02 | -0.03 | 6.033 | 1.03 | 0.02 | 228.23 | -0.04 | 676.20 | 35.89 | -0.46 | 24.48 | 171.33 | 1.57 | 11.49 |
| 13 | 2.24 | 0.85 | -0.01 | 5.156 | 0.49 | 0.00 | 4.644 | 0.46 | -0.02 | 257.94 | 0.73 | -598.31 | 52.44 | 1.20 | 19.45 | 173.89 | 1.00 | 5.88 |
| 14 | 1.50 | 0.94 | -0.01 | 5.544 | 0.63 | -0.02 | 5.767 | -0.27 | 0.44 | 215.12 | 0.30 | 1874.04 | 38.22 | 0.57 | 106.44 | 172.89 | 1.61 | 2.38 |
| 15 | 1.47 | 0.42 | -0.01 | 6.344 | 0.48 | 0.08 | 5.722 | 0.48 | 0.04 | 360.47 | 0.56 | 267.54 | 59.67 | 0.73 | -2.26 | 174.56 | 0.82 | -0.18 |
| 16 | 1.30 | 0.50 | -0.01 | 5.800 | 0.44 | 0.06 | 6.544 | 0.39 | 0.03 | 308.13 | 0.57 | 18228 | 49.89 | 0.73 | 12.82 | 169.78 | 1.16 | 1.29 |
| 17 | 2.81 | 3.32 | -0.01 | 6.956 | 0.65 | 0.00 | 7.533 | 0.78 | 0.03 | 373.61 | 0.64 | 983.01 | 51.56 | 0.64 | 41.32 | 176.11 | 0.82 | -0.36 |
| 18 | 4.13 | 0.83 | -0.01 | 7.122 | 1.09 | -0.01 | 7.800 | 1.08 | -0.02 | 619.62 | 1.06 | 445.27 | 82.89 | 1.08 | -1.99 | 172.89 | 0.54 | 9.62 |
| 19 | 2.58 | 2.27 | 0.00 | 7.044 | 0.74 | -0.03 | 7.533 | 0.87 | -0.03 | 583.87 | 0.75 | 297.46 | 80.00 | 0.50 | 11.60 | 174.22 | 1.42 | 12.86 |
| 20 | 1.37 | 0.70 | 0.02 | 4.733 | 1.05 | 0.04 | 5.189 | 1.05 | 0.09 | 430.95 | 0.95 | -732.74 | 86.56 | 0.74 | -5.79 | 174.89 | 0.47 | 2.67 |
| 21 | 2.20 | 1.03 | 0.05 | 6.867 | 0.22 | 0.12 | 6.400 | 0.35 | 0.21 | 248.00 | 0.35 | -69.02 | 37.56 | 0.52 | 33.70 | 175.22 | 0.80 | 2.82 |
| 22 | 2.42 | 0.98 | 0.03 | 6.567 | 0.77 | 0.04 | 7.033 | 0.96 | 0.05 | 274.48 | 0.30 | 323.84 | 40.56 | 0.19 | 105.73 | 177.00 | 0.46 | 0.61 |
| 23 | 1.99 | 1.03 | -0.01 | 6.367 | 0.90 | 0.03 | 5.867 | 0.69 | 0.11 | 258.45 | 0.46 | 1525.91 | 41.89 | 0.40 | 26.68 | 169.56 | 0.87 | 1.72 |
| 24 | 2.64 | 2.55 | 0.07 | 5.856 | 1.05 | 0.18 | 6.533 | 0.97 | 0.16 | 279.07 | 0.28 | 8091.55 | 44.89 | -0.07 | 146.82 | 177.67 | 0.39 | -0.45 |
| 25 | 1.46 | 1.11 | 0.02 | 7.122 | 0.45 | -0.01 | 7.689 | 0.53 | -0.01 | 318.59 | 1.26 | 5905.39 | 42.89 | 1.60 | 102.42 | 170.78 | 1.47 | 6.37 |
| 26 | 2.27 | 0.69 | 0.12 | 6.867 | 1.70 | 0.00 | 6.278 | 1.77 | 0.00 | 480.35 | 1.35 | -595.98 | 72.67 | 0.90 | 8.08 | 172.78 | 0.68 | 9.29 |
| 27 | 2.04 | 0.72 | 0.00 | 6.878 | 0.81 | 0.11 | 7.511 | 0.65 | 0.18 | 443.61 | 1.25 | 232.6 | 61.44 | 1.47 | -8.35 | 168.56 | 0.73 | 1.02 |
| 28 | 1.72 | 2.02 | -0.01 | 7.778 | 0.91 | 0.02 | 7.200 | 0.94 | 0.00 | 584.79 | 1.16 | 228.69 | 77.78 | 0.99 | -4.08 | 168.44 | 0.59 | 1.40 |
| 29 | 2.01 | 0.59 | -0.01 | 5.767 | -0.24 | 0.09 | 5.156 | -0.25 | 0.05 | 309.15 | 0.42 | -709.6 | 56.78 | 0.93 | 27.92 | 172.22 | 0.62 | -0.51 |
| 30 | 1.48 | 0.80 | 0.00 | 5.467 | 0.86 | -0.02 | 4.967 | 0.60 | -0.03 | 477.15 | 0.66 | -635.3 | 91.33 | 0.49 | -6.80 | 170.22 | 0.93 | 0.84 |
| 31 | 1.68 | 0.85 | -0.01 | 7.678 | 0.67 | 0.02 | 6.933 | 0.47 | 0.00 | 242.17 | 1.04 | -221.54 | 32.78 | 1.35 | 0.05 | 168.78 | 0.65 | 0.59 |
| 32 | 2.07 | 0.76 | -0.01 | 6.111 | 0.55 | 0.01 | 6.711 | 0.72 | 0.05 | 331.53 | -1.16 | 1592.11 | 36.68 | 1.53 | 5.39 | 169.22 | 0.97 | -0.37 |
| 33 | 1.69 | -0.12 | -0.01 | 6.211 | 0.23 | -0.02 | 6.744 | 0.45 | -0.02 | 323.59 | 1.33 | -778.16 | 49.78 | 1.97 | -8.99 | 168.22 | 0.78 | 8.91 |
| 34 | 2.07 | 0.87 | -0.01 | 8.333 | 0.43 | 0.16 | 8.733 | 0.45 | 0.19 | 527.34 | 0.94 | -427.29 | 61.67 | 0.91 | -9.42 | 177.56 | 1.06 | 0.94 |
| 35 | 1.60 | 0.25 | 0.04 | 7.422 | 0.59 | -0.02 | 6.756 | 0.64 | -0.01 | 245.87 | 1.3 | -915.51 | 34.44 | 1.73 | -9.11 | 168.56 | 0.82 | -0.18 |
| 36 | 2.22 | 0.73 | 0.04 | 5.111 | 0.21 | -0.03 | 5.567 | 0.31 | -0.03 | 254.71 | 0.19 | -337.72 | 47.67 | 0.21 | 4.60 | 179.56 | 0.80 | -0.50 |
| 37 | 2.40 | 0.43 | 0.04 | 6.667 | 0.71 | -0.03 | 7.200 | 0.78 | -0.03 | 285.19 | 1.53 | 1621.97 | 40.78 | 2.04 | 49.98 | 178.11 | 0.78 | -0.37 |
| 38 | 1.85 | 1.73 | -0.01 | 5.778 | 0.92 | -0.01 | 6.267 | 0.83 | -0.02 | 551.82 | 1.79 | 8493.57 | 90.78 | 2.31 | 198.50 | 178.78 | 0.43 | -0.52 |
| 39 | 2.25 | 0.82 | 0.00 | 5.544 | 1.33 | 0.00 | 4.956 | 1.16 | -0.02 | 497.49 | 1.29 | 5077.81 | 94.11 | 1.17 | 166.71 | 171.00 | 1.76 | 5.59 |
| 40 | 2.23 | 1.29 | 0.08 | 5.900 | 1.10 | 0.02 | 6.422 | 0.87 | 0.02 | 281.92 | 1.89 | 2035.2 | 54.33 | 0.48 | -0.51 | 173.00 | 1.02 | 5.14 |
| 41 | 1.97 | 0.67 | -0.01 | 5.967 | 1.21 | -0.03 | 6.456 | 1.15 | -0.02 | 243.24 | 1.18 | -792.29 | 38.67 | 1.56 | -5.14 | 172.11 | 1.07 | 0.97 |
| 42 | 2.96 | 1.73 | 0.02 | 7.100 | 1.50 | 0.01 | 7.567 | 1.34 | 0.00 | 277.63 | 1.37 | -866.18 | 37.33 | 1.54 | -8.53 | 176.45 | 1.19 | 7.62 |
| 43 | 2.13 | 0.26 | -0.01 | 6.578 | 1.03 | 0.01 | 7.011 | 1.06 | -0.02 | 288.30 | 1.25 | -849.23 | 42.00 | 1.51 | -10.34 | 175.67 | 0.73 | 1.56 |
| 44 | 1.78 | -0.09 | -0.01 | 7.933 | 1.12 | 0.04 | 7.467 | 0.98 | 0.03 | 355.41 | 0.93 | 3793.39 | 45.89 | 0.89 | 43.32 | 166.44 | 1.41 | 8.22 |
| 45 | 2.64 | 1.62 | 0.02 | 5.111 | 1.85 | -0.03 | 4.389 | 1.90 | -0.02 | 532.64 | 1.94 | 18494.94 | 110.56 | 1.62 | 837.28 | 168.00 | 0.93 | 4.08 |
| 46 | 2.00 | 1.15 | -0.01 | 6.433 | 1.17 | 0.00 | 6.789 | 1.26 | 0.00 | 360.00 | 0.42 | 89.86 | 54.22 | 0.01 | -3.69 | 169.22 | 0.95 | 2.10 |
| 47 | 2.05 | 1.07 | 0.01 | 5.411 | 1.26 | 0.02 | 5.756 | 1.14 | 0.03 | 189.99 | 0.39 | 7181.85 | 33.78 | 0.21 | 208.05 | 173.45 | 0.74 | 0.87 |
| 48 | 2.01 | 1.55 | 0.08 | 6.567 | 1.71 | -0.03 | 6.978 | 1.80 | -0.02 | 429.06 | 1.63 | 232.56 | 62.56 | 1.52 | 14.09 | 169.56 | 0.91 | 5.27 |
| 49 | 1.53 | 0.11 | 0.04 | 7.100 | 1.45 | 0.10 | 7.489 | 1.61 | 0.14 | 383.56 | 0.77 | -494.07 | 52.44 | 0.35 | 16.77 | 168.44 | 0.94 | -0.48 |
| 50 | 1.95 | 0.89 | 0.00 | 5.933 | 0.63 | -0.03 | 6.389 | 0.69 | -0.03 | 246.27 | 0.42 | 964.74 | 40.00 | 0.47 | 40.33 | 173.11 | 1.31 | 0.78 |
| 51 | 1.48 | 0.56 | 0.02 | 6.378 | 1.08 | 0.05 | 6.778 | 1.17 | 0.00 | 299.02 | 0.53 | 8781.31 | 45.11 | 0.30 | 175.99 | 169.33 | 1.19 | 5.81 |
| 52 | 1.70 | 0.88 | 0.00 | 7.422 | 1.15 | -0.02 | 7.800 | 1.23 | -0.03 | 277.19 | -0.21 | -612.88 | 36.56 | -0.70 | -2.86 | 167.89 | 0.96 | 2.10 |
| 53 | 2.19 | 1.71 | 0.01 | 7.611 | 1.51 | 0.06 | 7.989 | 1.49 | 0.04 | 353.88 | 0.48 | 4730.44 | 45.33 | 0.04 | 58.09 | 175.33 | 1.16 | -0.37 |
| 54 | 2.47 | 0.61 | 0.03 | 6.611 | 0.79 | 0.27 | 7.056 | 0.61 | 0.36 | 511.20 | 1.07 | 11118.5 | 74.22 | 1.22 | 78.15 | 171.89 | 0.26 | 15.54 |
| 55 | 1.94 | 0.76 | -0.01 | 6.478 | 1.20 | -0.01 | 7.089 | 1.09 | 0.14 | 401.36 | 0.56 | 4864.48 | 59.33 | 0.28 | 171.53 | 167.56 | 1.40 | 14.97 |
| 56 | 1.43 | 0.75 | -0.01 | 4.889 | 1.29 | 0.03 | 5.289 | 1.18 | -0.01 | 438.53 | 1.09 | 1518.03 | 85.78 | 0.83 | 31.21 | 167.11 | 0.81 | 0.20 |
| 57 | 2.12 | 0.62 | 0.02 | 5.522 | 1.67 | 0.00 | 5.933 | 1.57 | -0.03 | 358.95 | 0.94 | -878.43 | 62.33 | 0.53 | -10.38 | 172.44 | 0.74 | 0.87 |
| 58 | 2.26 | 1.32 | 0.16 | 6.211 | 0.55 | 0.08 | 6.811 | 0.57 | 0.06 | 623.92 | 1.45 | -652.33 | 95.56 | 1.92 | -7.61 | 170.11 | 1.02 | -0.42 |
| 59 | 2.30 | 1.82 | -0.01 | 6.067 | 0.80 | -0.03 | 6.433 | 0.70 | -0.02 | 407.43 | 0.89 | 6363.82 | 64.89 | 0.98 | 146.38 | 169.44 | 0.62 | 7.31 |
| 60 | 3.98 | 2.46 | 0.05 | 6.611 | 1.31 | 0.01 | 7.467 | 1.37 | 0.03 | 630.84 | 1.08 | -286.13 | 89.00 | 1.68 | -10.33 | 174.89 | 1.01 | 3.96 |
| 61 | 2.10 | 0.37 | -0.01 | 5.622 | 0.40 | -0.02 | 4.933 | 0.45 | -0.03 | 188.63 | 0.18 | 3749.71 | 35.89 | 0.20 | 165.57 | 174.67 | 0.09 | 2.77 |
| 62 | 1.88 | 1.06 | 0.00 | 6.467 | 0.53 | -0.03 | 5.811 | 0.45 | -0.03 | 364.38 | 0.40 | 84.41 | 59.11 | 0.39 | 15.76 | 172.00 | 1.23 | 2.47 |
| 63 | 2.29 | 0.85 | 0.00 | 4.678 | 1.43 | -0.02 | 4.200 | 1.19 | -0.03 | 405.93 | 1.06 | -570.87 | 91.11 | 0.84 | -7.31 | 169.33 | 1.13 | 7.59 |
| 64 | 2.06 | 0.56 | 0.00 | 4.589 | 1.34 | 0.01 | 4.089 | 1.13 | -0.03 | 391.72 | 1.25 | 1634.52 | 89.33 | 1.45 | 70.82 | 170.22 | 1.03 | 8.40 |
| 65 | 2.41 | 2.47 | 0.00 | 6.522 | 2.39 | -0.03 | 7.111 | 2.63 | -0.03 | 672.11 | 3.12 | -265.29 | 96.78 | 2.56 | 1.96 | 174.00 | 0.95 | 0.30 |
| 66 | 2.54 | 2.67 | -0.01 | 6.756 | 0.51 | 0.02 | 7.344 | 0.55 | -0.01 | 671.01 | 1.25 | -881.96 | 94.89 | 1.08 | -3.09 | 175.45 | 0.48 | 14.17 |
| 67 | 1.71 | 1.77 | 0.03 | 5.178 | 1.59 | 0.00 | 5.589 | 1.61 | 0.03 | 285.13 | 0.72 | 1209.82 | 52.67 | 0.32 | 37.86 | 169.44 | 1.32 | 7.50 |
| 68 | 2.60 | 0.73 | 0.02 | 6.611 | 0.99 | 0.28 | 7.044 | 1.10 | 0.23 | 560.86 | 1.29 | 1346.82 | 81.78 | 1.28 | -10.24 | 175.78 | 1.09 | 2.29 |
| 69 | 1.60 | 0.55 | 0.04 | 6.500 | 0.79 | -0.02 | 6.867 | 0.92 | -0.03 | 354.08 | 0.90 | 165.00 | 52.78 | 1.00 | 14.65 | 161.89 | 0.60 | 0.74 |
| 70 | 3.20 | 0.87 | 0.04 | 8.222 | 0.46 | 0.03 | 8.856 | 0.77 | 0.12 | 733.17 | 0.43 | 1964.47 | 95.67 | 1.06 | -3.41 | 180.33 | 0.69 | 16.40 |
| 71 | 1.73 | 0.59 | 0.00 | 5.178 | 1.13 | 0.11 | 5.522 | 1.07 | 0.12 | 190.89 | 0.43 | 3853.79 | 35.44 | 0.29 | 96.78 | 165.11 | 1.40 | 1.07 |
| 72 | 2.79 | 0.42 | -0.01 | 7.367 | 1.22 | 0.01 | 6.856 | 1.09 | 0.03 | 314.46 | 0.37 | -526.18 | 44.11 | 0.06 | -9.38 | 164.22 | 0.53 | 3.24 |
| 73 | 1.90 | 0.30 | -0.01 | 5.644 | 0.54 | -0.03 | 6.133 | 0.70 | -0.02 | 246.22 | 0.33 | 152.85 | 41.67 | 0.29 | 17.35 | 157.00 | 1.54 | 1.21 |
| 74 | 1.98 | 0.58 | 0.01 | 5.656 | 0.75 | -0.03 | 6.067 | 0.85 | -0.03 | 445.88 | 0.90 | -877.54 | 75.78 | 0.95 | -10.26 | 165.56 | 0.72 | 8.12 |
| 75 | 2.09 | 0.92 | 0.00 | 4.767 | 1.10 | -0.02 | 5.178 | 1.12 | -0.03 | 203.41 | 0.65 | 29.52 | 40.56 | 0.77 | 21.95 | 170.00 | 0.97 | 8.82 |
| 76 | 2.03 | 0.33 | 0.00 | 5.044 | 1.39 | 0.01 | 5.789 | 1.42 | 0.03 | 260.31 | 0.63 | 1364.47 | 47.89 | 0.34 | 36.40 | 169.44 | 2.04 | 6.14 |
| 77 | 1.61 | 1.67 | -0.01 | 4.856 | 1.05 | -0.03 | 5.300 | 1.19 | -0.01 | 398.47 | 1.19 | 326.28 | 77.89 | 1.38 | 53.44 | 166.67 | 0.99 | 2.77 |
| 78 | 1.55 | 0.18 | 0.04 | 5.278 | 1.12 | -0.03 | 5.689 | 1.11 | -0.03 | 272.68 | 0.77 | -331.96 | 49.44 | 0.83 | 8.92 | 163.22 | 1.57 | 0.12 |
| 79 | 1.50 | 1.47 | 0.00 | 5.789 | 1.15 | -0.02 | 6.244 | 1.44 | 0.01 | 542.34 | 2.58 | 233.11 | 88.67 | 3.31 | 68.90 | 159.22 | 0.63 | 8.85 |
| 80 | 1.52 | 0.75 | 0.05 | 6.711 | 0.99 | 0.16 | 7.200 | 1.00 | 0.11 | 617.53 | 2.07 | 7652.38 | 87.89 | 2.41 | 60.87 | 165.22 | 1.37 | 2.66 |
| 81 | 1.96 | 0.55 | 0.00 | 5.311 | 1.95 | -0.03 | 5.856 | 2.16 | -0.02 | 230.12 | 1.31 | 7637.19 | 87.78 | 1.39 | 9.83 | 165.89 | 0.90 | 6.74 |
| 82 | 2.24 | 2.05 | 0.13 | 6.244 | 0.24 | -0.02 | 7.011 | 0.39 | -0.02 | 511.86 | 1.12 | 6704.4 | 77.00 | 1.54 | 138.31 | 175.11 | 0.94 | -0.48 |
| 83 | 1.69 | 2.39 | -0.01 | 6.867 | 0.81 | 0.00 | 7.200 | 0.96 | -0.02 | 283.37 | 0.39 | -513.56 | 40.22 | 0.62 | -10.00 | 172.33 | 0.87 | 4.18 |
| 84 | 2.08 | 0.66 | -0.01 | 6.456 | 0.89 | 0.04 | 6.956 | 0.93 | 0.01 | 449.89 | 0.86 | -895.88 | 66.89 | 0.73 | -8.62 | 168.56 | 1.37 | 2.67 |
| 85 | 2.19 | 0.65 | -0.01 | 7.800 | 1.39 | 0.29 | 8.433 | 1.45 | 0.31 | 528.51 | 1.29 | 4417.03 | 64.67 | 1.00 | 7.09 | 173.33 | 0.79 | -0.17 |
| 86 | 1.84 | 0.77 | -0.01 | 6.800 | 1.01 | -0.03 | 7.178 | 1.08 | -0.01 | 379.59 | 1.24 | 1333.9 | 54.00 | 1.35 | 21.91 | 167.22 | 0.64 | 1.33 |
| 87 | 2.09 | 0.70 | 0.00 | 7.256 | 0.61 | 0.12 | 7.833 | 0.94 | 0.23 | 421.69 | 0.27 | 1209.79 | 56.00 | 0.03 | -4.52 | 173.56 | 0.74 | 5.65 |
| 88 | 2.76 | 2.65 | 0.01 | 7.044 | 1.33 | 0.00 | 7.511 | 1.21 | -0.03 | 636.29 | 1.73 | 4983.4 | 86.78 | 1.57 | 65.83 | 173.89 | 0.79 | 1.95 |
| 89 | 2.38 | 1.25 | -0.01 | 5.867 | 0.60 | 0.18 | 6.600 | 1.09 | 0.07 | 437.91 | 0.82 | -320.24 | 70.11 | 0.86 | 55.70 | 169.56 | 0.49 | 0.80 |
| 90 | 2.51 | 1.35 | -0.01 | 6.989 | 1.06 | 0.01 | 7.822 | 0.41 | 0.17 | 467.48 | 0.94 | 6384.84 | 62.78 | 0.96 | 63.94 | 167.33 | 1.02 | 5.14 |
| 91 | 1.89 | 0.42 | 0.00 | 6.856 | 0.68 | -0.01 | 7.256 | 0.62 | -0.03 | 294.62 | 0.30 | 4551.04 | 41.89 | 0.24 | 106.99 | 165.11 | 0.73 | 4.41 |
| 92 | 1.30 | 0.77 | 0.00 | 5.578 | 1.55 | -0.03 | 5.867 | 1.57 | -0.03 | 283.08 | 0.96 | 5337.06 | 49.22 | 0.87 | 164.23 | 161.78 | 0.78 | -0.37 |
| 93 | 1.33 | 0.72 | -0.01 | 6.778 | 1.11 | -0.03 | 6.389 | 1.05 | 0.00 | 395.18 | 1.24 | -162.95 | 59.44 | 1.32 | -4.21 | 164.56 | 1.31 | 3.95 |
| 94 | 1.46 | 0.68 | -0.01 | 5.122 | 1.30 | -0.02 | 5.422 | 1.25 | 0.00 | 447.56 | 1.53 | -489.15 | 84.11 | 1.72 | 31.85 | 168.33 | 1.88 | 2.10 |
| 95 | 1.92 | 0.92 | 0.01 | 6.256 | 1.05 | -0.03 | 6.678 | 1.13 | -0.03 | 433.81 | 1.09 | -749.43 | 66.67 | 1.04 | -8.83 | 173.00 | 2.21 | 2.00 |
| 96 | 1.67 | 0.13 | -0.01 | 6.556 | 1.57 | -0.02 | 6.967 | 1.45 | 0.04 | 391.38 | 1.15 | -413.16 | 57.44 | 0.93 | 14.06 | 169.56 | 2.55 | 0.22 |
| 97 | 2.05 | 0.88 | -0.01 | 5.444 | 0.88 | 0.00 | 5.967 | 0.98 | -0.02 | 306.67 | 0.65 | -185.48 | 53.56 | 0.62 | 25.61 | 166.56 | 1.41 | 0.49 |
| 98 | 3.22 | 2.22 | 0.42 | 6.311 | 1.07 | 0.27 | 6.967 | 0.89 | 0.19 | 449.85 | 1.06 | -655.97 | 67.44 | 1.14 | -3.17 | 172.89 | 2.43 | 9.09 |
| 99 | 2.40 | 2.62 | 0.20 | 6.956 | 0.90 | -0.03 | 7.333 | 0.94 | -0.03 | 484.25 | 1.06 | -764.95 | 67.56 | 0.99 | 6.97 | 166.00 | 0.91 | 8.22 |
| 100 | 2.70 | 0.77 | 0.06 | 7.267 | 1.25 | 0.14 | 7.822 | 1.02 | 0.15 | 580.38 | 1.22 | -764.76 | 76.67 | 1.01 | -4.60 | 166.22 | 1.17 | -0.11 |
| 101 | 2.40 | 2.00 | 0.00 | 6.478 | 1.45 | 0.01 | 6.911 | 1.46 | 0.04 | 278.81 | 0.85 | 1834.78 | 41.11 | 0.77 | 30.13 | 165.67 | 1.01 | -0.19 |
| 102 | 4.19 | 0.83 | -0.01 | 7.978 | 0.56 | -0.03 | 7.322 | 0.75 | 0.03 | 857.06 | 1.02 | -331.73 | 103.44 | 1.05 | 6.61 | 170.44 | 1.55 | 54.04 |
| 103 | 1.85 | 0.89 | 0.01 | 6.889 | 0.97 | -0.01 | 7.244 | 1.06 | 0.03 | 440.47 | 1.24 | -648.78 | 62.00 | 1.23 | 8.30 | 161.00 | 0.84 | 3.56 |
| 104 | 2.23 | 0.85 | -0.01 | 7.556 | 1.04 | 0.00 | 8.111 | 1.17 | 0.00 | 622.97 | 1.62 | 1238.89 | 79.22 | 1.43 | 52.51 | 164.22 | 1.00 | 0.18 |
| 105 | 1.64 | 0.98 | 0.00 | 7.233 | 0.90 | 0.06 | 6.644 | 0.86 | 0.03 | 539.61 | 1.37 | 1083.48 | 77.33 | 1.50 | 2.54 | 161.78 | 1.19 | 11.70 |
| 106 | 3.09 | 1.16 | 0.18 | 5.711 | 1.26 | -0.02 | 6.522 | 1.36 | -0.02 | 510.78 | 2.31 | -829.86 | 82.33 | 2.86 | 6.20 | 171.22 | 0.95 | 2.10 |
| 107 | 3.25 | 2.19 | 0.06 | 7.700 | 1.53 | -0.02 | 7.067 | 1.39 | 0.06 | 478.26 | 0.96 | 597.62 | 64.44 | 0.48 | 5.49 | 172.11 | 0.75 | 11.48 |
| 108 | 2.19 | 1.17 | 0.11 | 8.667 | 1.81 | -0.03 | 7.222 | 1.42 | -0.02 | 407.74 | 1.70 | 1727.53 | 50.78 | 1.58 | 26.60 | 165.44 | 1.26 | -0.45 |
| 109 | 2.33 | 1.99 | 0.06 | 6.156 | 1.46 | 0.02 | 6.822 | 1.53 | -0.01 | 509.91 | 1.73 | 6198.37 | 77.67 | 1.72 | 96.53 | 171.33 | 1.29 | 3.54 |
| 110 | 2.20 | 0.99 | -0.01 | 6.467 | 0.54 | 0.03 | 6.900 | 0.58 | 0.05 | 452.18 | 0.97 | -916.91 | 67.44 | 1.11 | -0.85 | 170.11 | 1.19 | 0.82 |
| 111 | 2.47 | 2.26 | -0.01 | 6.889 | 1.30 | -0.02 | 7.744 | 1.23 | 0.00 | 579.39 | 2.12 | 2571.56 | 78.44 | 2.21 | 60.25 | 167.11 | 1.22 | 0.51 |
| 112 | 1.77 | 1.02 | 0.00 | 7.656 | 1.20 | -0.03 | 8.200 | 1.12 | -0.03 | 386.13 | 0.33 | -698.78 | 48.67 | -0.06 | -8.73 | 163.89 | 0.87 | 9.40 |
| 113 | 1.49 | 0.58 | 0.04 | 6.711 | 0.54 | -0.03 | 7.100 | 0.56 | -0.02 | 521.66 | 1.60 | -101.66 | 75.11 | 2.04 | 2.99 | 165.33 | 0.71 | 28.18 |
| 114 | 2.12 | 0.57 | 0.01 | 6.989 | 1.60 | -0.01 | 7.611 | 1.15 | -0.01 | 590.74 | 1.35 | -905.81 | 80.56 | 0.93 | -9.20 | 169.00 | 0.40 | 23.85 |
| 115 | 1.39 | 0.42 | -0.01 | 7.056 | 0.24 | -0.03 | 7.478 | 0.28 | -0.03 | 386.83 | 0.46 | 147.3 | 54.33 | 0.35 | 14.03 | 164.78 | 0.84 | 10.65 |
| 116 | 2.27 | 1.09 | -0.01 | 6.733 | 0.91 | -0.02 | 7.478 | 0.72 | 0.05 | 506.38 | 1.55 | 8034.04 | 70.67 | 1.79 | 287.40 | 173.33 | 1.00 | 7.52 |
| 117 | 1.84 | 0.54 | 0.02 | 7.144 | 0.89 | -0.03 | 7.622 | 0.91 | -0.03 | 369.57 | 0.79 | 1858.96 | 51.67 | 0.72 | 36.86 | 167.33 | 1.76 | 14.58 |
| 118 | 2.52 | 0.59 | 0.01 | 8.833 | 0.92 | 0.11 | 7.800 | 1.50 | 0.17 | 832.93 | 0.70 | 8360.4 | 99.44 | 1.72 | 31.85 | 178.56 | 1.53 | 22.31 |
| 119 | 2.03 | -0.07 | -0.01 | 8.267 | 0.48 | -0.03 | 6.967 | 0.32 | 0.07 | 675.12 | 1.15 | 1612.81 | 88.44 | 1.27 | 9.73 | 170.56 | 1.39 | -0.21 |
| 120 | 2.53 | 1.61 | 0.09 | 8.478 | 0.69 | 0.20 | 7.067 | 1.00 | 0.01 | 688.93 | 0.31 | 6425.63 | 99.33 | 1.14 | 17.18 | 180.22 | 1.69 | 62.37 |
|  | 2.10 |  |  | 6.53 |  |  | 6.76 |  |  | 416.43 |  | 62.71 |  |  |  | 170.58 |  | |

Supplemental Table S2(B): Estimation of mean and stability parameters for plant height, number of main branches, leaf length and breadth and days to 50% flowering.

|  | Plant Height (cm) | | | No. of Main Branch | | | Leaf Length (cm) | | | Leaf Breadth (cm) | | | Days to 50% Flowering | | |
| --- | --- | --- | --- | --- | --- | --- | --- | --- | --- | --- | --- | --- | --- | --- | --- |
| Line No. | Mean (µ) | βi | σ²di | Mean (µ) | βi | σ²di | Mean (µ) | βi | σ²di | Mean (µ) | βi | σ²di | Mean (µ) | βi | σ²di |
| 1 | 61.54 | 0.56 | 2.22 | 4.44 | 3.01 | 0.11 | 9.61 | 1.89 | 0.46 | 5.58 | 2.15 | 0.21 | 76.89 | 0.93 | 4.05 |
| 2 | 57.70 | 0.99 | -5.14 | 5.44 | 0.27 | -0.12 | 9.38 | 1.79 | 0.01 | 5.49 | 1.87 | 0.04 | 73.00 | 0.99 | 10.36 |
| 3 | 67.23 | 0.25 | 9.10 | 3.44 | 0.93 | 1.11 | 8.91 | 1.56 | -0.03 | 5.08 | 1.74 | 0.07 | 70.78 | 0.75 | 5.79 |
| 4 | 58.42 | 0.67 | -4.01 | 5.33 | 0.44 | 0.68 | 8.71 | 1.24 | -0.12 | 4.87 | 1.35 | 0.01 | 72.67 | 0.59 | -0.11 |
| 5 | 67.80 | 1.56 | 9.45 | 4.89 | 0.98 | -0.01 | 8.31 | 0.37 | 0.66 | 4.70 | 0.47 | 0.13 | 81.11 | 0.59 | -0.51 |
| 6 | 52.26 | 0.79 | -5.10 | 4.00 | 2.08 | 0.17 | 8.51 | 0.77 | -0.04 | 4.80 | 0.86 | -0.06 | 74.44 | 0.64 | 1.38 |
| 7 | 62.63 | -0.54 | 5.96 | 4.78 | -0.98 | -0.01 | 8.69 | 1.14 | 0.14 | 4.87 | 1.05 | 0.23 | 77.67 | 0.61 | 0.81 |
| 8 | 60.20 | 0.64 | 0.56 | 3.44 | 1.75 | -0.05 | 9.12 | 1.58 | 0.00 | 5.21 | 1.68 | -0.09 | 73.44 | 1.00 | 6.09 |
| 9 | 75.47 | 0.77 | -4.89 | 4.33 | 3.78 | 0.06 | 9.94 | 2.43 | 0.24 | 5.84 | 2.18 | 0.25 | 66.33 | 0.55 | 3.24 |
| 10 | 67.44 | 1.05 | -1.04 | 4.56 | 3.50 | 0.38 | 9.38 | 0.58 | -0.06 | 5.49 | 0.72 | 0.19 | 70.67 | 1.04 | 3.72 |
| 11 | 56.22 | 0.86 | -2.16 | 3.00 | -1.04 | -0.10 | 7.37 | 0.36 | 0.01 | 4.91 | 0.13 | 0.56 | 67.89 | 0.61 | -0.23 |
| 12 | 65.22 | 0.58 | 7.00 | 3.22 | 0.77 | -0.19 | 8.49 | 0.83 | 0.61 | 4.71 | 0.80 | 0.20 | 69.11 | 1.58 | 7.70 |
| 13 | 52.49 | 0.73 | 13.05 | 2.89 | 0.98 | -0.01 | 7.39 | 0.05 | 3.97 | 4.06 | 0.27 | 1.57 | 73.78 | 1.05 | 1.21 |
| 14 | 51.31 | 1.75 | -2.13 | 3.78 | 4.43 | 1.91 | 8.19 | 0.41 | 0.26 | 4.58 | 0.43 | 0.64 | 70.56 | 1.35 | 5.99 |
| 15 | 59.42 | 1.13 | -0.07 | 4.56 | 3.06 | -0.16 | 7.83 | -0.92 | 3.30 | 4.42 | -0.55 | 1.93 | 73.00 | 0.58 | -0.60 |
| 16 | 50.80 | 0.78 | 7.92 | 4.11 | -3.89 | 0.70 | 7.27 | 0.61 | 1.03 | 3.90 | 0.61 | 0.36 | 70.22 | 1.35 | 5.99 |
| 17 | 73.20 | 0.15 | 55.43 | 3.89 | 2.24 | 0.16 | 8.39 | 0.99 | 0.50 | 4.67 | 1.09 | 0.33 | 74.56 | 0.69 | -0.43 |
| 18 | 65.12 | 1.16 | -2.65 | 5.22 | 2.02 | -0.17 | 8.91 | 0.86 | 0.03 | 5.09 | 0.95 | 0.27 | 70.67 | 0.79 | 12.81 |
| 19 | 66.22 | -0.68 | 0.77 | 3.78 | 2.35 | 0.54 | 8.37 | 2.18 | 0.07 | 4.60 | 2.21 | 0.72 | 73.22 | 0.59 | 0.63 |
| 20 | 62.44 | 1.58 | 26.22 | 4.67 | 3.56 | -0.19 | 9.62 | 0.72 | -0.06 | 5.69 | 0.69 | 0.07 | 71.56 | 1.08 | 1.85 |
| 21 | 62.50 | 2.40 | 29.98 | 3.44 | 1.75 | -0.05 | 8.47 | 1.38 | -0.10 | 4.69 | 1.45 | -0.09 | 75.11 | 1.19 | -0.05 |
| 22 | 61.28 | 1.48 | 2.47 | 5.00 | 2.30 | -0.17 | 7.90 | 1.42 | 0.22 | 4.22 | 1.52 | 0.53 | 75.89 | 0.41 | 0.58 |
| 23 | 58.59 | 0.55 | 29.33 | 3.00 | 0.22 | 0.03 | 7.34 | 1.04 | -0.07 | 3.63 | 0.88 | 0.12 | 71.56 | 0.67 | 2.04 |
| 24 | 59.60 | 0.97 | 0.55 | 2.78 | 0.49 | -0.14 | 7.69 | 0.82 | 0.31 | 3.96 | 0.82 | 0.38 | 76.11 | 0.60 | -0.69 |
| 25 | 57.77 | 1.99 | 0.51 | 4.22 | 3.28 | -0.11 | 9.27 | 1.82 | -0.07 | 5.51 | 1.91 | -0.07 | 71.22 | 1.52 | -0.66 |
| 26 | 70.21 | 0.73 | 58.10 | 3.89 | 2.68 | 2.11 | 9.10 | 1.53 | 0.76 | 5.30 | 1.71 | 0.75 | 70.56 | 1.11 | 9.48 |
| 27 | 74.62 | 0.61 | 4.84 | 3.22 | 0.16 | 1.22 | 8.02 | 1.24 | -0.01 | 4.32 | 1.28 | 0.06 | 66.78 | 0.85 | 5.70 |
| 28 | 62.14 | 0.29 | 8.12 | 4.56 | 1.59 | 0.46 | 8.08 | 1.97 | 0.58 | 4.40 | 1.96 | 0.81 | 70.33 | 0.69 | 1.27 |
| 29 | 61.98 | 1.72 | 36.70 | 4.89 | 2.24 | 0.16 | 9.32 | 0.69 | -0.02 | 5.48 | 0.76 | -0.05 | 74.78 | 0.39 | 1.22 |
| 30 | 51.02 | 0.22 | 7.51 | 3.89 | -0.93 | 1.11 | 5.80 | 0.37 | 0.19 | 3.82 | -1.72 | 0.80 | 68.22 | 1.03 | 7.25 |
| 31 | 54.18 | 1.34 | 78.97 | 4.44 | 1.31 | 0.12 | 7.86 | 0.82 | -0.04 | 4.16 | 0.79 | -0.08 | 66.44 | 0.90 | 3.16 |
| 32 | 59.61 | 0.10 | 9.90 | 4.22 | -0.06 | 0.33 | 8.36 | 0.45 | -0.11 | 4.57 | 0.47 | -0.05 | 67.89 | 1.07 | 1.55 |
| 33 | 51.93 | 0.31 | 14.94 | 5.22 | -0.49 | -0.14 | 7.80 | 1.12 | -0.02 | 4.22 | 1.14 | -0.09 | 66.11 | 0.84 | 6.72 |
| 34 | 57.73 | 1.99 | 12.15 | 5.78 | 1.53 | -0.18 | 8.21 | 1.55 | -0.10 | 4.53 | 1.64 | -0.08 | 76.89 | 0.78 | -0.58 |
| 35 | 54.76 | -0.76 | 23.25 | 3.67 | -0.22 | 0.03 | 8.32 | 1.35 | 0.12 | 4.29 | 0.77 | 0.51 | 65.89 | 0.85 | 3.32 |
| 36 | 51.17 | 2.28 | 13.95 | 3.89 | 2.90 | 3.74 | 7.66 | 0.65 | 0.24 | 3.89 | 0.64 | -0.09 | 79.33 | 0.33 | -0.63 |
| 37 | 57.33 | 0.14 | 46.01 | 4.56 | 2.24 | 0.16 | 7.26 | -0.42 | 0.97 | 3.90 | 0.09 | 0.29 | 78.00 | 0.39 | -0.41 |
| 38 | 59.63 | -0.55 | 32.94 | 4.00 | -2.08 | 0.17 | 9.36 | 0.28 | -0.11 | 5.62 | 0.29 | -0.06 | 77.67 | 0.47 | -0.02 |
| 39 | 63.38 | 0.58 | 1.87 | 3.22 | -0.06 | 0.33 | 5.54 | 0.19 | 0.05 | 2.71 | 0.15 | -0.04 | 79.33 | 0.75 | 5.31 |
| 40 | 55.41 | 2.25 | 38.46 | 2.78 | 2.19 | 1.52 | 8.04 | 0.98 | 0.28 | 4.40 | 0.94 | 0.18 | 72.67 | 0.60 | 2.80 |
| 41 | 54.23 | 0.40 | 9.18 | 3.33 | 0.44 | 0.68 | 8.03 | 1.06 | 0.30 | 4.40 | 1.06 | 0.11 | 71.22 | 0.59 | 1.49 |
| 42 | 60.66 | 2.32 | 56.45 | 4.11 | 3.61 | 0.28 | 8.78 | 1.75 | -0.12 | 4.98 | 1.96 | -0.07 | 79.78 | 0.55 | 2.65 |
| 43 | 61.97 | 0.72 | -0.77 | 3.33 | -0.22 | 0.03 | 8.57 | 1.20 | 0.03 | 4.87 | 1.33 | -0.02 | 74.56 | 0.47 | -0.55 |
| 44 | 62.80 | 1.52 | -4.92 | 3.11 | 1.53 | -0.18 | 7.93 | 2.20 | 0.19 | 4.48 | 2.19 | 0.12 | 67.78 | 0.87 | 0.37 |
| 45 | 61.03 | 0.84 | 13.06 | 3.67 | -0.22 | 0.03 | 5.91 | 0.73 | -0.04 | 3.17 | 0.60 | 0.13 | 67.00 | 1.10 | 1.11 |
| 46 | 57.62 | 1.86 | 3.25 | 4.78 | 5.31 | -0.02 | 9.53 | 1.04 | 0.84 | 5.63 | 1.15 | 1.03 | 70.56 | 0.91 | 0.86 |
| 47 | 50.01 | 1.52 | 99.98 | 4.67 | 3.34 | 0.00 | 7.82 | -0.62 | 0.85 | 4.12 | -0.43 | 0.38 | 71.56 | 0.59 | 1.49 |
| 48 | 62.72 | 1.11 | -5.13 | 5.22 | 0.77 | -0.19 | 10.39 | 0.49 | 3.94 | 6.13 | 0.19 | 3.02 | 68.78 | 1.12 | 0.49 |
| 49 | 66.94 | 0.90 | -5.10 | 4.22 | 3.50 | 0.38 | 8.22 | 2.08 | -0.07 | 4.50 | 1.81 | -0.07 | 66.67 | 1.00 | -0.20 |
| 50 | 61.51 | 1.44 | 32.56 | 3.56 | 1.37 | 1.43 | 8.28 | 0.89 | -0.02 | 4.37 | 0.76 | -0.06 | 69.89 | 1.08 | 1.85 |
| 51 | 54.87 | 2.66 | 36.30 | 4.56 | 1.20 | 0.60 | 9.38 | 0.99 | 0.51 | 5.56 | 1.00 | 0.38 | 65.44 | 0.83 | 4.39 |
| 52 | 59.76 | 1.29 | 22.52 | 3.67 | 1.86 | 0.94 | 9.92 | 0.97 | 0.56 | 6.06 | 1.02 | 0.35 | 69.22 | 1.29 | 10.49 |
| 53 | 55.03 | 1.76 | 9.81 | 3.78 | 2.35 | 0.54 | 8.18 | 0.98 | 0.72 | 4.54 | 0.91 | 0.42 | 74.00 | 0.58 | -0.60 |
| 54 | 58.60 | 0.64 | 73.35 | 3.89 | 0.33 | 0.76 | 9.41 | 0.05 | -0.10 | 5.60 | 0.06 | -0.08 | 72.89 | 0.71 | 6.41 |
| 55 | 63.34 | 1.38 | -5.13 | 4.00 | -0.22 | 0.03 | 9.33 | 1.45 | 0.63 | 5.48 | 1.50 | 0.64 | 68.44 | 1.45 | 14.88 |
| 56 | 58.40 | 0.58 | 35.22 | 4.22 | 2.63 | 1.04 | 8.84 | 0.88 | -0.12 | 5.00 | 0.98 | -0.09 | 67.11 | 1.31 | 2.94 |
| 57 | 55.76 | 1.49 | 269.64 | 4.33 | 2.08 | 0.17 | 7.30 | 0.27 | -0.10 | 3.80 | 0.45 | -0.08 | 71.00 | 0.77 | 0.54 |
| 58 | 56.93 | 4.34 | 53.34 | 4.56 | 0.98 | -0.01 | 10.46 | 0.40 | 0.36 | 6.32 | 0.33 | -0.03 | 70.67 | 1.02 | 1.44 |
| 59 | 51.27 | 1.81 | 4.92 | 4.56 | 2.24 | 0.16 | 79.16 | 0.11 | -0.11 | 5.28 | -0.17 | -0.04 | 77.67 | 0.81 | 9.60 |
| 60 | 67.11 | 0.80 | 35.25 | 4.67 | 0.66 | 1.76 | 10.50 | 2.22 | -0.07 | 5.89 | 1.32 | 0.42 | 75.22 | 1.98 | 15.83 |
| 61 | 56.11 | -0.21 | 43.07 | 4.56 | -3.23 | 0.83 | 7.84 | 0.27 | -0.06 | 4.20 | 0.40 | 0.02 | 79.33 | 0.77 | 0.54 |
| 62 | 60.16 | 0.89 | 57.41 | 3.89 | 0.11 | 1.88 | 7.82 | 0.80 | -0.12 | 4.18 | 1.01 | -0.02 | 70.22 | 1.27 | 5.12 |
| 63 | 60.30 | 0.91 | 56.25 | 4.78 | 1.31 | 0.12 | 5.97 | 0.06 | -0.01 | 3.24 | 0.35 | 0.11 | 68.44 | 1.39 | 3.63 |
| 64 | 60.90 | 1.78 | 62.58 | 3.00 | -0.22 | 0.03 | 6.06 | 0.26 | -0.10 | 3.10 | 0.15 | -0.01 | 69.11 | 1.39 | 3.63 |
| 65 | 65.61 | -0.11 | 24.03 | 3.44 | 1.10 | 0.85 | 9.71 | 0.68 | -0.08 | 5.87 | 0.69 | 0.05 | 80.00 | 1.08 | 4.23 |
| 66 | 68.00 | 0.44 | 3.63 | 4.11 | 0.71 | 0.27 | 9.93 | 0.96 | -0.09 | 6.12 | 1.03 | 0.03 | 83.78 | 0.67 | 10.49 |
| 67 | 51.60 | 2.05 | 163.60 | 4.22 | -0.71 | 0.27 | 7.66 | 0.63 | 0.06 | 4.01 | 0.78 | 0.08 | 70.22 | 1.71 | 13.38 |
| 68 | 62.47 | 0.63 | -4.20 | 4.00 | 0.66 | 1.76 | 9.50 | 1.05 | 0.36 | 5.69 | 1.07 | 0.55 | 78.44 | 0.67 | 4.93 |
| 69 | 68.13 | 1.46 | 30.38 | 3.67 | 0.22 | 0.03 | 10.32 | 1.06 | 0.66 | 6.22 | 0.88 | 1.11 | 67.67 | 1.43 | 1.79 |
| 70 | 71.09 | 1.14 | -4.85 | 4.56 | 1.24 | 0.16 | 10.39 | 0.49 | 0.02 | 6.50 | 0.54 | -0.06 | 90.44 | 1.05 | 8.11 |
| 71 | 57.19 | 0.95 | 44.59 | 3.56 | 0.11 | 1.88 | 8.41 | 1.29 | -0.10 | 4.67 | 1.47 | -0.09 | 67.89 | 1.14 | 0.01 |
| 72 | 64.76 | 0.74 | 42.44 | 4.33 | 3.12 | 0.62 | 8.81 | 1.74 | -0.12 | 4.97 | 2.07 | -0.08 | 74.67 | 0.75 | 5.31 |
| 73 | 42.78 | 1.14 | 8.42 | 4.44 | 0.27 | -0.12 | 7.93 | 1.69 | 0.40 | 4.03 | 1.22 | -0.08 | 65.78 | 1.71 | 2.72 |
| 74 | 60.87 | 0.78 | -2.17 | 4.44 | 3.01 | 0.11 | 8.36 | 0.58 | 1.65 | 4.53 | -0.12 | 0.15 | 74.00 | 0.79 | 12.81 |
| 75 | 53.18 | 1.07 | 38.84 | 3.67 | -1.26 | -0.16 | 7.30 | -0.68 | -0.07 | 3.99 | -0.11 | -0.09 | 76.00 | 0.69 | 1.27 |
| 76 | 51.86 | 0.81 | -5.14 | 3.11 | 0.27 | -0.12 | 8.69 | 0.73 | -0.12 | 4.83 | 0.61 | -0.09 | 75.67 | 0.88 | 4.22 |
| 77 | 60.30 | 0.19 | 2.28 | 3.67 | -1.26 | -0.16 | 9.63 | 1.00 | 0.08 | 5.81 | 1.08 | 0.05 | 67.56 | 0.88 | -0.59 |
| 78 | 55.86 | 1.31 | 3.80 | 4.44 | -0.98 | -0.01 | 10.60 | 1.73 | -0.05 | 6.53 | 1.44 | -0.07 | 65.11 | 0.85 | -0.68 |
| 79 | 47.28 | 0.56 | 14.20 | 4.11 | 0.27 | -0.12 | 7.47 | 0.25 | 0.88 | 3.89 | 0.64 | 0.28 | 65.33 | 0.79 | 12.82 |
| 80 | 58.53 | 0.84 | 76.17 | 3.00 | -1.26 | -0.16 | 9.29 | 1.63 | -0.11 | 5.30 | 1.61 | -0.08 | 68.67 | 0.84 | -0.02 |
| 81 | 53.22 | 2.49 | 18.49 | 4.67 | 3.12 | 0.62 | 8.33 | 0.62 | 0.17 | 4.58 | 0.78 | 0.02 | 72.44 | 0.71 | 3.09 |
| 82 | 56.78 | 1.34 | -2.25 | 5.00 | 1.48 | 0.21 | 6.88 | 0.64 | -0.10 | 3.56 | 0.73 | 0.19 | 80.11 | 0.77 | -0.54 |
| 83 | 49.82 | 2.00 | 2.76 | 3.89 | 0.98 | -0.01 | 9.28 | 1.33 | -0.07 | 5.46 | 1.46 | 0.00 | 74.78 | 1.32 | 10.50 |
| 84 | 61.86 | 1.06 | 3.46 | 4.22 | 1.20 | 0.60 | 9.69 | 1.78 | -0.04 | 5.69 | 1.60 | 0.14 | 78.89 | 0.99 | 1.32 |
| 85 | 62.19 | 0.82 | -4.17 | 4.00 | 0.22 | 0.03 | 9.20 | 1.43 | -0.12 | 5.40 | 1.54 | -0.09 | 80.44 | 0.43 | -0.59 |
| 86 | 56.54 | 1.03 | 52.97 | 4.44 | 1.31 | 0.12 | 10.14 | 1.44 | -0.07 | 6.18 | 1.39 | -0.09 | 72.56 | 1.76 | 9.48 |
| 87 | 63.41 | 0.90 | 18.27 | 4.67 | 0.82 | 0.40 | 8.69 | 0.97 | -0.11 | 4.72 | 0.70 | -0.05 | 82.44 | 1.31 | 13.84 |
| 88 | 62.68 | 0.97 | 41.74 | 4.00 | 2.08 | 0.17 | 9.36 | 0.55 | -0.12 | 5.36 | 0.19 | 0.09 | 82.67 | 0.48 | -0.57 |
| 89 | 53.77 | 2.13 | 12.79 | 3.78 | 3.23 | 0.83 | 9.30 | 0.94 | 0.15 | 5.40 | 0.87 | 0.22 | 79.22 | 0.71 | -0.69 |
| 90 | 66.18 | 1.20 | 70.46 | 3.56 | -0.88 | 2.01 | 9.38 | 1.34 | 0.11 | 5.56 | 1.45 | 0.16 | 77.89 | 0.63 | 7.37 |
| 91 | 59.24 | 0.89 | 46.81 | 4.11 | 1.31 | 0.12 | 9.76 | 1.44 | -0.11 | 6.01 | 1.60 | 0.04 | 72.33 | 1.31 | 6.20 |
| 92 | 51.81 | 1.36 | 23.41 | 4.22 | -0.11 | 3.44 | 8.16 | 0.70 | 0.15 | 4.24 | 0.55 | 0.34 | 68.56 | 1.21 | -0.39 |
| 93 | 57.21 | 0.66 | 129.18 | 4.78 | -0.16 | 1.22 | 7.68 | 0.39 | 0.72 | 4.00 | 0.47 | 0.90 | 72.89 | 1.60 | 6.30 |
| 94 | 63.94 | 2.21 | 40.80 | 3.78 | -1.42 | 1.64 | 9.62 | 1.62 | -0.12 | 5.71 | 1.75 | -0.09 | 75.22 | 2.08 | -0.58 |
| 95 | 61.21 | 0.94 | 20.82 | 3.67 | 0.82 | 0.40 | 9.73 | 1.20 | -0.02 | 6.08 | 1.19 | -0.02 | 75.89 | 1.69 | -0.65 |
| 96 | 63.69 | 2.03 | 15.03 | 3.89 | 3.50 | 0.38 | 9.06 | 1.61 | -0.10 | 5.28 | 1.83 | -0.08 | 73.78 | 1.83 | -0.58 |
| 97 | 53.93 | 1.42 | 5.06 | 4.00 | 2.52 | -0.08 | 8.73 | 1.44 | -0.06 | 5.20 | 1.20 | 0.18 | 78.33 | 0.98 | 7.47 |
| 98 | 59.47 | 1.25 | 52.31 | 4.78 | 1.75 | -0.05 | 8.36 | 1.96 | 0.30 | 4.52 | 2.22 | 0.32 | 76.56 | 0.73 | 4.14 |
| 99 | 58.73 | 0.81 | -4.92 | 3.44 | -2.24 | 0.16 | 7.66 | 0.60 | 0.18 | 6.00 | 0.70 | 0.48 | 69.33 | 0.82 | 8.16 |
| 100 | 60.28 | 1.03 | 52.49 | 4.00 | 2.74 | 0.45 | 9.66 | 1.28 | 0.13 | 5.79 | 1.35 | 0.09 | 78.11 | 0.34 | -0.45 |
| 101 | 60.53 | -0.42 | 17.44 | 4.11 | -2.46 | 0.92 | 7.62 | 0.53 | 1.32 | 3.98 | 0.62 | 1.23 | 76.00 | 1.17 | 0.41 |
| 102 | 60.91 | -0.18 | 45.01 | 4.22 | 0.33 | 0.76 | 8.54 | 1.52 | -0.10 | 4.73 | 1.76 | -0.04 | 76.89 | 1.14 | 0.01 |
| 103 | 58.41 | 2.06 | 1.33 | 3.89 | 0.16 | 1.22 | 9.27 | 1.01 | -0.12 | 5.46 | 1.11 | -0.09 | 68.89 | 1.17 | 2.44 |
| 104 | 65.33 | 0.06 | 8.29 | 3.67 | 1.26 | -0.16 | 9.03 | 1.68 | 1.03 | 5.21 | 2.01 | 1.07 | 74.67 | 0.84 | -0.02 |
| 105 | 61.86 | 1.20 | 3.22 | 4.44 | 1.31 | 0.12 | 8.18 | 1.99 | -0.10 | 4.46 | 2.16 | -0.09 | 66.33 | 0.83 | 11.15 |
| 106 | 60.40 | 0.13 | -3.68 | 4.78 | -1.20 | 0.60 | 8.93 | -0.13 | 0.82 | 5.17 | -0.15 | 0.94 | 79.00 | 0.94 | 1.44 |
| 107 | 64.16 | 1.23 | -3.45 | 3.22 | 0.55 | 0.07 | 10.60 | 0.89 | -0.08 | 6.58 | 0.72 | -0.08 | 82.11 | 1.00 | 8.99 |
| 108 | 65.50 | -0.14 | 51.53 | 3.89 | -0.49 | -0.14 | 8.79 | 1.23 | -0.09 | 4.84 | 1.25 | -0.08 | 75.44 | 1.47 | 2.51 |
| 109 | 58.72 | 0.60 | 12.32 | 4.11 | 0.71 | 0.27 | 8.87 | 0.50 | 0.62 | 5.07 | 0.55 | 0.75 | 82.44 | 1.31 | -0.17 |
| 110 | 62.39 | 0.80 | -5.09 | 4.67 | 1.26 | -0.16 | 10.59 | 2.07 | 4.34 | 6.29 | 1.28 | 2.51 | 81.67 | 1.43 | 1.61 |
| 111 | 63.98 | 0.78 | 11.04 | 5.00 | 1.48 | 0.21 | 7.84 | 1.56 | 0.32 | 4.11 | 1.77 | 0.18 | 78.78 | 0.61 | -0.43 |
| 112 | 63.23 | 1.38 | 161.70 | 3.78 | 0.06 | 0.33 | 9.66 | 2.00 | 0.07 | 5.64 | 1.99 | 0.03 | 72.78 | 1.19 | 4.58 |
| 113 | 65.01 | 0.18 | 42.89 | 4.11 | 0.71 | 0.27 | 9.76 | 0.92 | -0.08 | 5.93 | 0.94 | -0.07 | 74.33 | 1.36 | 14.13 |
| 114 | 63.56 | 1.31 | -4.93 | 5.44 | 1.75 | -0.05 | 9.30 | 1.49 | 0.05 | 5.48 | 1.67 | 0.04 | 76.78 | 1.35 | 65.36 |
| 115 | 69.61 | 0.87 | -3.02 | 3.89 | -1.75 | -0.05 | 9.72 | 0.33 | 0.76 | 5.83 | 0.35 | 0.84 | 74.22 | 1.08 | 16.30 |
| 116 | 71.23 | 0.94 | -4.17 | 5.22 | -0.49 | -0.14 | 9.76 | 1.06 | -0.10 | 5.80 | 0.93 | -0.09 | 83.89 | 1.44 | 8.05 |
| 117 | 58.81 | 0.81 | -3.49 | 3.67 | 0.22 | 0.03 | 8.74 | 1.61 | 0.17 | 5.21 | 1.74 | -0.05 | 76.22 | 1.79 | 4.40 |
| 118 | 68.63 | -0.11 | -3.47 | 4.89 | 2.02 | -0.17 | 9.61 | 0.83 | 0.19 | 5.76 | 0.88 | 0.23 | 87.89 | 2.11 | 25.46 |
| 119 | 72.24 | 0.71 | -5.04 | 5.00 | 2.52 | -0.08 | 9.11 | 0.86 | -0.11 | 5.31 | 0.98 | -0.07 | 73.89 | 1.62 | -0.45 |
| 120 | 65.84 | 1.04 | -0.55 | 5.00 | 0.44 | 0.68 | 8.60 | 0.73 | 0.39 | 4.83 | 0.84 | 0.27 | 91.11 | 1.95 | 46.96 |
|  | 60.29 |  |  | 4.13 |  |  | 8.67 |  |  | 4.95 |  |  | 73.80 |  |  |

Supplemental Table S3(A): Mean and IPCA score for capsaicin content across three environments based on AMMI model

| Line No. | Mean (Capsaicin content %) | | | AMMI | | | Adj  Mean | PCA I | Var Index | G*E ResSS |
| --- | --- | --- | --- | --- | --- | --- | --- | --- | --- | --- |
|  | Env 1 | Env 2 | Env 3 | Env 1 | Env 2 | Env 3 |  |  |  |  |
| 1 | 2.137 | 1.997 | 2.090 | 2.142 | 1.920 | 2.162 | 2.074 | -0.010 | -0.013 | 0.011 |
| 2 | 1.993 | 1.733 | 1.757 | 2.001 | 1.626 | 1.857 | 1.828 | 0.082 | -0.259 | 0.022 |
| 3 | 1.893 | 1.620 | 1.853 | 1.893 | 1.618 | 1.856 | 1.789 | 0.023 | -0.298 | 0.000 |
| 4 | 1.910 | 1.873 | 1.963 | 1.916 | 1.791 | 2.040 | 1.916 | -0.069 | -0.172 | 0.013 |
| 5 | 3.233 | 2.620 | 3.040 | 3.226 | 2.723 | 2.944 | 2.964 | 0.160 | 0.877 | 0.020 |
| 6 | 1.863 | 1.633 | 1.750 | 1.868 | 1.571 | 1.808 | 1.749 | 0.035 | -0.338 | 0.007 |
| 7 | 1.533 | 1.460 | 1.837 | 1.529 | 1.522 | 1.779 | 1.610 | -0.139 | -0.477 | 0.007 |
| 8 | 1.707 | 1.323 | 1.760 | 1.700 | 1.426 | 1.664 | 1.597 | 0.021 | -0.491 | 0.020 |
| 9 | 1.970 | 1.923 | 1.990 | 1.976 | 1.830 | 2.077 | 1.961 | -0.055 | -0.126 | 0.016 |
| 10 | 1.937 | 1.897 | 1.913 | 1.945 | 1.778 | 2.024 | 1.916 | -0.043 | -0.172 | 0.026 |
| 11 | 2.003 | 1.967 | 2.040 | 2.010 | 1.876 | 2.124 | 2.003 | -0.063 | -0.084 | 0.015 |
| 12 | 1.657 | 1.567 | 1.580 | 1.665 | 1.448 | 1.690 | 1.601 | -0.013 | -0.486 | 0.026 |
| 13 | 2.287 | 2.100 | 2.320 | 2.288 | 2.088 | 2.331 | 2.236 | -0.023 | 0.148 | 0.000 |
| 14 | 1.517 | 1.347 | 1.623 | 1.516 | 1.362 | 1.609 | 1.496 | -0.051 | -0.592 | 0.000 |
| 15 | 1.513 | 1.407 | 1.500 | 1.519 | 1.328 | 1.573 | 1.473 | -0.029 | -0.614 | 0.011 |
| 16 | 1.303 | 1.217 | 1.367 | 1.307 | 1.166 | 1.414 | 1.296 | -0.059 | -0.792 | 0.005 |
| 17 | 3.093 | 2.277 | 3.050 | 3.074 | 2.563 | 2.784 | 2.807 | 0.165 | 0.719 | 0.153 |
| 18 | 4.160 | 4.020 | 4.200 | 4.162 | 3.986 | 4.232 | 4.127 | -0.038 | 2.039 | 0.002 |
| 19 | 2.813 | 2.213 | 2.703 | 2.804 | 2.351 | 2.576 | 2.577 | 0.130 | 0.489 | 0.035 |
| 20 | 1.300 | 1.263 | 1.557 | 1.299 | 1.282 | 1.539 | 1.373 | -0.134 | -0.714 | 0.001 |
| 21 | 2.103 | 2.033 | 2.453 | 2.098 | 2.116 | 2.376 | 2.197 | -0.155 | 0.109 | 0.013 |
| 22 | 2.347 | 2.260 | 2.640 | 2.342 | 2.324 | 2.581 | 2.416 | -0.133 | 0.328 | 0.008 |
| 23 | 2.120 | 1.823 | 2.023 | 2.121 | 1.805 | 2.040 | 1.989 | 0.047 | -0.098 | 0.001 |
| 24 | 3.047 | 2.230 | 2.643 | 3.039 | 2.337 | 2.544 | 2.640 | 0.280 | 0.553 | 0.021 |
| 25 | 1.423 | 1.283 | 1.673 | 1.418 | 1.354 | 1.608 | 1.460 | -0.105 | -0.627 | 0.009 |
| 26 | 2.063 | 2.163 | 2.583 | 2.058 | 2.240 | 2.512 | 2.270 | -0.254 | 0.183 | 0.011 |
| 27 | 2.027 | 1.930 | 2.177 | 2.027 | 1.928 | 2.179 | 2.044 | -0.084 | -0.043 | 0.000 |
| 28 | 1.827 | 1.393 | 1.927 | 1.816 | 1.546 | 1.784 | 1.716 | 0.019 | -0.372 | 0.044 |
| 29 | 2.107 | 1.913 | 2.003 | 2.112 | 1.837 | 2.075 | 2.008 | 0.022 | -0.079 | 0.011 |
| 30 | 1.457 | 1.357 | 1.637 | 1.456 | 1.371 | 1.623 | 1.483 | -0.093 | -0.604 | 0.000 |
| 31 | 1.723 | 1.540 | 1.763 | 1.724 | 1.529 | 1.773 | 1.676 | -0.026 | -0.412 | 0.000 |
| 32 | 2.140 | 1.943 | 2.113 | 2.143 | 1.907 | 2.148 | 2.066 | -0.001 | -0.022 | 0.003 |
| 33 | 1.633 | 1.707 | 1.720 | 1.642 | 1.582 | 1.836 | 1.687 | -0.108 | -0.401 | 0.029 |
| 34 | 2.140 | 1.930 | 2.137 | 2.141 | 1.912 | 2.153 | 2.069 | -0.005 | -0.018 | 0.001 |
| 35 | 1.467 | 1.567 | 1.780 | 1.468 | 1.541 | 1.804 | 1.604 | -0.187 | -0.483 | 0.001 |
| 36 | 2.113 | 2.103 | 2.440 | 2.111 | 2.143 | 2.403 | 2.219 | -0.163 | 0.132 | 0.003 |
| 37 | 2.263 | 2.330 | 2.597 | 2.263 | 2.332 | 2.595 | 2.397 | -0.185 | 0.309 | 0.000 |
| 38 | 1.940 | 1.573 | 2.033 | 1.932 | 1.687 | 1.927 | 1.849 | 0.004 | -0.238 | 0.024 |
| 39 | 2.253 | 2.123 | 2.383 | 2.253 | 2.129 | 2.378 | 2.253 | -0.069 | 0.166 | 0.000 |
| 40 | 2.117 | 2.023 | 2.543 | 2.107 | 2.157 | 2.419 | 2.228 | -0.174 | 0.141 | 0.033 |
| 41 | 2.027 | 1.863 | 2.020 | 2.030 | 1.819 | 2.062 | 1.970 | -0.016 | -0.117 | 0.004 |
| 42 | 3.213 | 2.680 | 2.980 | 3.211 | 2.720 | 2.943 | 2.958 | 0.152 | 0.871 | 0.003 |
| 43 | 2.177 | 2.083 | 2.117 | 2.184 | 1.975 | 2.218 | 2.126 | -0.017 | 0.038 | 0.022 |
| 44 | 1.773 | 1.790 | 1.763 | 1.783 | 1.648 | 1.896 | 1.776 | -0.062 | -0.312 | 0.038 |
| 45 | 2.647 | 2.380 | 2.887 | 2.638 | 2.513 | 2.762 | 2.638 | -0.069 | 0.551 | 0.033 |
| 46 | 2.063 | 1.813 | 2.113 | 2.061 | 1.843 | 2.085 | 1.997 | -0.012 | -0.091 | 0.002 |
| 47 | 2.020 | 1.880 | 2.250 | 2.016 | 1.941 | 2.193 | 2.050 | -0.099 | -0.037 | 0.007 |
| 48 | 2.343 | 1.763 | 1.930 | 2.345 | 1.739 | 1.953 | 2.012 | 0.222 | -0.075 | 0.001 |
| 49 | 1.383 | 1.517 | 1.700 | 1.386 | 1.475 | 1.739 | 1.533 | -0.197 | -0.554 | 0.003 |
| 50 | 1.963 | 1.810 | 2.083 | 1.962 | 1.823 | 2.071 | 1.952 | -0.060 | -0.135 | 0.000 |
| 51 | 1.393 | 1.387 | 1.647 | 1.393 | 1.388 | 1.646 | 1.476 | -0.141 | -0.612 | 0.000 |
| 52 | 1.840 | 1.563 | 1.710 | 1.843 | 1.518 | 1.752 | 1.704 | 0.052 | -0.383 | 0.004 |
| 53 | 2.210 | 1.913 | 2.433 | 2.200 | 2.054 | 2.302 | 2.186 | -0.056 | 0.098 | 0.037 |
| 54 | 2.370 | 2.377 | 2.670 | 2.369 | 2.394 | 2.654 | 2.472 | -0.159 | 0.385 | 0.001 |
| 55 | 2.027 | 1.820 | 1.980 | 2.030 | 1.779 | 2.019 | 1.942 | 0.008 | -0.145 | 0.003 |
| 56 | 1.493 | 1.313 | 1.493 | 1.496 | 1.281 | 1.523 | 1.433 | -0.014 | -0.654 | 0.002 |
| 57 | 2.303 | 2.023 | 2.043 | 2.311 | 1.915 | 2.144 | 2.123 | 0.095 | 0.036 | 0.022 |
| 58 | 2.653 | 2.047 | 2.080 | 2.660 | 1.957 | 2.164 | 2.260 | 0.280 | 0.173 | 0.015 |
| 59 | 2.450 | 2.013 | 2.450 | 2.443 | 2.118 | 2.352 | 2.304 | 0.052 | 0.217 | 0.021 |
| 60 | 2.933 | 2.167 | 2.583 | 2.926 | 2.273 | 2.484 | 2.561 | 0.040 | 0.474 | 0.021 |
| 61 | 2.167 | 2.037 | 2.083 | 2.174 | 1.936 | 2.177 | 2.096 | 0.000 | 0.008 | 0.019 |
| 62 | 1.903 | 1.713 | 2.030 | 1.901 | 1.749 | 1.996 | 1.882 | -0.052 | -0.205 | 0.002 |
| 63 | 2.283 | 2.150 | 2.423 | 2.282 | 2.162 | 2.412 | 2.286 | -0.071 | 0.198 | 0.000 |
| 64 | 2.023 | 1.973 | 2.190 | 2.025 | 1.954 | 2.208 | 2.062 | -0.101 | -0.025 | 0.001 |
| 65 | 2.533 | 2.013 | 2.677 | 2.518 | 2.234 | 2.471 | 2.408 | 0.028 | 0.321 | 0.091 |
| 66 | 2.710 | 2.117 | 2.800 | 2.694 | 2.350 | 2.583 | 2.542 | 0.064 | 0.455 | 0.102 |
| 67 | 1.707 | 1.430 | 1.997 | 1.695 | 1.594 | 1.844 | 1.711 | -0.082 | -0.376 | 0.050 |
| 68 | 2.790 | 2.480 | 2.523 | 2.797 | 2.384 | 2.612 | 2.598 | 0.105 | 0.511 | 0.017 |
| 69 | 1.477 | 1.513 | 1.807 | 1.476 | 1.529 | 1.792 | 1.599 | -0.176 | -0.488 | 0.000 |
| 70 | 3.203 | 3.100 | 2.887 | 3.219 | 2.869 | 3.102 | 3.063 | 0.048 | 0.976 | 0.100 |
| 71 | 1.853 | 1.633 | 1.700 | 1.859 | 1.546 | 1.781 | 1.729 | 0.045 | -0.358 | 0.014 |
| 72 | 3.043 | 2.933 | 3.027 | 3.049 | 2.855 | 3.099 | 3.001 | -0.027 | 0.914 | 0.011 |
| 73 | 1.897 | 1.857 | 1.960 | 1.902 | 1.781 | 2.030 | 1.904 | -0.071 | -0.183 | 0.011 |
| 74 | 1.910 | 1.887 | 2.137 | 1.910 | 1.883 | 2.140 | 1.978 | -0.128 | -0.109 | 0.000 |
| 75 | 2.107 | 1.947 | 2.227 | 2.106 | 1.963 | 2.211 | 2.093 | -0.058 | 0.006 | 0.001 |
| 76 | 1.997 | 1.977 | 2.113 | 2.001 | 1.917 | 2.169 | 2.029 | -0.093 | -0.058 | 0.007 |
| 77 | 1.753 | 1.343 | 1.733 | 1.748 | 1.424 | 1.658 | 1.610 | 0.052 | -0.477 | 0.012 |
| 78 | 1.413 | 1.527 | 1.723 | 1.416 | 1.492 | 1.756 | 1.554 | -0.190 | -0.533 | 0.002 |
| 79 | 1.530 | 1.267 | 1.707 | 1.523 | 1.367 | 1.613 | 1.501 | -0.049 | -0.586 | 0.019 |
| 80 | 1.403 | 1.400 | 1.750 | 1.400 | 1.446 | 1.707 | 1.518 | -0.171 | -0.569 | 0.004 |
| 81 | 2.067 | 1.873 | 1.947 | 2.073 | 1.788 | 2.026 | 1.962 | 0.028 | -0.125 | 0.014 |
| 82 | 2.670 | 1.910 | 2.140 | 2.669 | 1.924 | 2.127 | 2.240 | 0.306 | 0.153 | 0.000 |
| 83 | 1.883 | 1.303 | 1.870 | 1.871 | 1.478 | 1.707 | 1.686 | 0.094 | -0.402 | 0.057 |
| 84 | 2.157 | 1.973 | 2.107 | 2.160 | 1.918 | 2.158 | 2.079 | 0.003 | -0.008 | 0.006 |
| 85 | 2.203 | 2.083 | 2.273 | 2.205 | 2.054 | 2.301 | 2.187 | -0.052 | 0.099 | 0.002 |
| 86 | 1.920 | 1.713 | 1.877 | 1.923 | 1.674 | 1.914 | 1.837 | 0.007 | -0.251 | 0.003 |
| 87 | 2.223 | 1.973 | 2.060 | 2.229 | 1.897 | 2.131 | 2.086 | 0.056 | -0.002 | 0.011 |
| 88 | 2.863 | 2.337 | 3.077 | 2.846 | 2.596 | 2.836 | 2.759 | 0.007 | 0.672 | 0.126 |
| 89 | 2.430 | 2.177 | 2.520 | 2.426 | 2.228 | 2.472 | 2.376 | -0.024 | 0.288 | 0.005 |
| 90 | 2.583 | 2.300 | 2.660 | 2.579 | 2.361 | 2.603 | 2.514 | -0.012 | 0.427 | 0.007 |
| 91 | 1.853 | 1.827 | 1.997 | 1.856 | 1.784 | 2.037 | 1.892 | -0.100 | -0.195 | 0.003 |
| 92 | 1.437 | 1.177 | 1.287 | 1.441 | 1.112 | 1.347 | 1.300 | 0.055 | -0.787 | 0.008 |
| 93 | 1.437 | 1.217 | 1.343 | 1.441 | 1.159 | 1.397 | 1.332 | 0.026 | -0.755 | 0.006 |
| 94 | 1.563 | 1.347 | 1.457 | 1.568 | 1.281 | 1.518 | 1.456 | 0.029 | -0.632 | 0.008 |
| 95 | 1.897 | 1.770 | 2.080 | 1.895 | 1.800 | 2.052 | 1.916 | -0.087 | -0.172 | 0.002 |
| 96 | 1.707 | 1.647 | 1.650 | 1.715 | 1.522 | 1.766 | 1.668 | -0.027 | -0.419 | 0.029 |
| 97 | 2.163 | 1.913 | 2.087 | 2.166 | 1.880 | 2.118 | 2.054 | 0.028 | -0.033 | 0.002 |
| 98 | 3.863 | 2.863 | 2.943 | 3.867 | 2.811 | 2.992 | 3.223 | 0.044 | 1.136 | 0.005 |
| 99 | 2.933 | 1.980 | 2.290 | 2.929 | 2.041 | 2.234 | 2.401 | 0.393 | 0.314 | 0.007 |
| 100 | 2.947 | 2.570 | 2.570 | 2.955 | 2.455 | 2.677 | 2.696 | 0.058 | 0.608 | 0.025 |
| 101 | 2.623 | 2.083 | 2.503 | 2.616 | 2.184 | 2.410 | 2.403 | 0.118 | 0.316 | 0.019 |
| 102 | 4.223 | 4.183 | 4.157 | 4.233 | 4.043 | 4.287 | 4.188 | -0.029 | 2.101 | 0.037 |
| 103 | 1.830 | 1.710 | 2.013 | 1.828 | 1.737 | 1.988 | 1.851 | -0.089 | -0.236 | 0.001 |
| 104 | 2.323 | 2.093 | 2.273 | 2.325 | 2.063 | 2.302 | 2.230 | 0.015 | 0.143 | 0.002 |
| 105 | 1.800 | 1.483 | 1.637 | 1.803 | 1.443 | 1.675 | 1.640 | 0.074 | -0.447 | 0.003 |
| 106 | 3.487 | 2.900 | 2.877 | 3.495 | 2.781 | 2.987 | 3.088 | 0.287 | 1.001 | 0.026 |
| 107 | 3.300 | 2.593 | 2.940 | 3.295 | 2.663 | 2.875 | 2.944 | 0.238 | 0.857 | 0.009 |
| 108 | 2.040 | 2.010 | 2.533 | 2.031 | 2.143 | 2.409 | 2.194 | -0.212 | 0.107 | 0.033 |
| 109 | 2.667 | 2.007 | 2.303 | 2.664 | 2.050 | 2.263 | 2.326 | 0.227 | 0.238 | 0.003 |
| 110 | 2.303 | 2.037 | 2.247 | 2.304 | 2.022 | 2.260 | 2.196 | 0.026 | 0.108 | 0.000 |
| 111 | 2.657 | 2.107 | 2.640 | 2.646 | 2.264 | 2.494 | 2.468 | 0.087 | 0.381 | 0.046 |
| 112 | 1.797 | 1.610 | 1.910 | 1.795 | 1.638 | 1.884 | 1.772 | -0.049 | -0.315 | 0.001 |
| 113 | 1.700 | 1.400 | 1.383 | 1.709 | 1.274 | 1.501 | 1.494 | 0.118 | -0.593 | 0.030 |
| 114 | 2.057 | 2.030 | 2.273 | 2.057 | 2.024 | 2.279 | 2.120 | -0.124 | 0.033 | 0.000 |
| 115 | 1.470 | 1.327 | 1.387 | 1.476 | 1.233 | 1.474 | 1.394 | 0.003 | -0.693 | 0.016 |
| 116 | 2.407 | 2.100 | 2.317 | 2.407 | 2.090 | 2.326 | 2.274 | 0.047 | 0.187 | 0.000 |
| 117 | 1.770 | 1.757 | 2.000 | 1.770 | 1.750 | 2.006 | 1.842 | -0.131 | -0.245 | 0.000 |
| 118 | 2.667 | 2.427 | 2.470 | 2.673 | 2.328 | 2.561 | 2.521 | 0.044 | 0.434 | 0.018 |
| 119 | 1.970 | 2.040 | 2.077 | 1.978 | 1.927 | 2.182 | 2.029 | -0.113 | -0.058 | 0.024 |
| 120 | 2.890 | 2.273 | 2.437 | 2.892 | 2.249 | 2.460 | 2.533 | 0.044 | 0.446 | 0.001 |

Supplemental Table S3(B): Mean and IPCA score for fruit yield per plant across three environments based on AMMI model

| Line No. | Mean (Fruit yield per plant (g)) | | | AMMI | | | Adj  Mean | PCA I | Var Index | G*E  Res SS |
| --- | --- | --- | --- | --- | --- | --- | --- | --- | --- | --- |
|  | Env 1 | Env 2 | Env 3 | Env 1 | Env 2 | Env 3 |  |  |  |  |
| 1 | 404.93 | 344.68 | 516.90 | 428.48 | 355.71 | 482.33 | 422.17 | 0.39 | 9.15 | 1871.39 |
| 2 | 389.80 | 380.90 | 526.85 | 417.14 | 393.70 | 486.71 | 432.52 | 1.71 | 19.50 | 2521.98 |
| 3 | 302.92 | 304.97 | 367.80 | 297.34 | 302.36 | 375.99 | 325.23 | 2.48 | -87.79 | 105.01 |
| 4 | 444.67 | 417.43 | 367.80 | 381.48 | 387.85 | 460.57 | 409.97 | 2.52 | -3.05 | 13472.71 |
| 5 | 588.92 | 503.40 | 693.13 | 612.60 | 514.49 | 658.36 | 595.15 | -0.29 | 182.13 | 1892.69 |
| 6 | 328.22 | 305.42 | 433.93 | 343.85 | 312.74 | 410.98 | 355.86 | 1.51 | -57.16 | 824.84 |
| 7 | 250.28 | 257.37 | 409.42 | 285.01 | 273.63 | 358.43 | 305.69 | 2.04 | -107.33 | 4069.80 |
| 8 | 435.60 | 371.60 | 532.62 | 453.16 | 379.82 | 506.83 | 446.61 | 0.37 | 33.59 | 1041.17 |
| 9 | 379.35 | 340.17 | 545.75 | 423.66 | 360.91 | 480.70 | 421.76 | 0.66 | 8.74 | 6624.98 |
| 10 | 368.62 | 350.60 | 463.45 | 378.85 | 355.39 | 448.42 | 394.22 | 1.71 | -18.80 | 353.55 |
| 11 | 262.17 | 215.63 | 330.97 | 265.03 | 216.98 | 326.76 | 269.59 | 1.05 | -143.43 | 27.72 |
| 12 | 195.55 | 242.75 | 246.40 | 177.60 | 234.35 | 272.75 | 228.23 | 3.87 | -184.79 | 1087.02 |
| 13 | 281.55 | 198.60 | 293.68 | 264.82 | 190.77 | 318.25 | 257.94 | 0.35 | -155.08 | 945.11 |
| 14 | 176.33 | 208.03 | 261.00 | 175.25 | 207.53 | 262.59 | 215.12 | 3.21 | -197.90 | 3.97 |
| 15 | 395.37 | 308.37 | 377.67 | 366.21 | 294.72 | 420.47 | 360.47 | 0.42 | -52.55 | 2867.98 |
| 16 | 278.03 | 278.85 | 367.50 | 283.32 | 281.33 | 359.74 | 308.13 | 2.29 | -104.89 | 94.36 |
| 17 | 346.60 | 338.32 | 435.92 | 353.08 | 341.35 | 426.40 | 373.61 | 2.03 | -39.41 | 141.93 |
| 18 | 644.93 | 529.07 | 684.87 | 644.86 | 529.03 | 684.98 | 619.62 | -0.77 | 206.60 | 0.02 |
| 19 | 565.22 | 538.27 | 648.12 | 571.50 | 541.21 | 638.89 | 583.87 | 1.53 | 170.85 | 133.23 |
| 20 | 431.98 | 364.12 | 496.75 | 436.05 | 366.02 | 490.77 | 430.95 | 0.46 | 17.93 | 55.92 |
| 21 | 228.92 | 230.48 | 284.60 | 219.40 | 226.03 | 298.57 | 248.00 | 2.52 | -165.02 | 305.45 |
| 22 | 227.62 | 270.65 | 325.18 | 230.57 | 272.03 | 320.84 | 274.48 | 3.46 | -138.54 | 29.47 |
| 23 | 224.43 | 238.42 | 312.50 | 227.28 | 239.75 | 308.31 | 258.45 | 2.68 | -154.57 | 27.45 |
| 24 | 206.12 | 285.63 | 345.45 | 222.18 | 293.16 | 321.86 | 279.07 | 4.25 | -133.95 | 870.86 |
| 25 | 401.12 | 200.63 | 354.02 | 374.92 | 188.37 | 392.48 | 318.59 | -2.67 | -94.43 | 2316.30 |
| 26 | 511.95 | 374.15 | 554.95 | 516.25 | 376.16 | 548.63 | 480.35 | -1.42 | 67.33 | 62.49 |
| 27 | 486.70 | 340.92 | 503.20 | 480.58 | 338.05 | 512.18 | 443.61 | -1.49 | 30.59 | 126.32 |
| 28 | 572.20 | 507.93 | 674.23 | 591.98 | 517.20 | 645.19 | 584.79 | 0.33 | 171.77 | 1320.92 |
| 29 | 326.10 | 273.68 | 327.67 | 300.53 | 261.71 | 365.20 | 309.15 | 1.30 | -103.87 | 2206.12 |
| 30 | 499.08 | 422.90 | 509.47 | 480.65 | 414.27 | 536.53 | 477.15 | 0.56 | 64.13 | 1146.90 |
| 31 | 276.58 | 157.05 | 292.87 | 266.73 | 152.44 | 307.33 | 242.17 | -0.73 | -170.85 | 327.70 |
| 32 | 276.50 | 432.40 | 285.70 | 225.35 | 408.46 | 360.79 | 331.53 | 7.27 | -81.49 | 8828.49 |
| 33 | 330.92 | 227.62 | 412.23 | 347.10 | 235.19 | 388.47 | 323.59 | -0.66 | -89.43 | 884.20 |
| 34 | 521.40 | 463.52 | 597.10 | 528.84 | 467.00 | 586.17 | 527.34 | 0.68 | 114.32 | 186.92 |
| 35 | 260.93 | 149.77 | 326.92 | 271.54 | 154.73 | 311.34 | 245.87 | -0.80 | -167.15 | 379.70 |
| 36 | 276.48 | 233.87 | 253.78 | 239.00 | 216.32 | 308.82 | 254.71 | 1.73 | -158.31 | 4741.84 |
| 37 | 345.12 | 156.78 | 353.67 | 341.44 | 155.06 | 359.06 | 285.19 | -2.67 | -127.83 | 45.54 |
| 38 | 652.63 | 390.33 | 612.48 | 638.04 | 383.50 | 633.92 | 551.82 | -4.50 | 138.80 | 719.26 |
| 39 | 576.27 | 378.57 | 537.63 | 553.37 | 367.84 | 571.26 | 497.49 | -2.64 | 84.47 | 1769.94 |
| 40 | 349.65 | 125.10 | 371.00 | 356.57 | 128.34 | 360.84 | 281.92 | -3.79 | -131.10 | 161.58 |
| 41 | 267.20 | 152.07 | 310.45 | 268.47 | 152.66 | 308.59 | 243.24 | -0.77 | -169.78 | 5.42 |
| 42 | 289.13 | 177.68 | 366.08 | 304.55 | 184.90 | 343.45 | 277.63 | -0.87 | -135.39 | 802.18 |
| 43 | 297.38 | 197.72 | 369.80 | 309.19 | 203.25 | 352.46 | 288.30 | -0.50 | -124.72 | 470.74 |
| 44 | 422.55 | 265.83 | 377.83 | 391.32 | 251.21 | 423.69 | 355.41 | -1.42 | -57.61 | 3292.18 |
| 45 | 669.48 | 347.57 | 580.87 | 642.07 | 334.73 | 621.12 | 532.64 | -5.92 | 119.62 | 2536.13 |
| 46 | 390.87 | 319.70 | 369.43 | 357.89 | 304.26 | 417.84 | 360.00 | 0.90 | -53.02 | 3669.02 |
| 47 | 122.40 | 186.90 | 260.67 | 140.08 | 195.18 | 234.70 | 189.99 | 3.83 | -223.03 | 1055.28 |
| 48 | 477.05 | 297.52 | 512.62 | 483.91 | 300.73 | 502.55 | 429.06 | -2.58 | 16.04 | 158.77 |
| 49 | 409.90 | 320.18 | 420.60 | 393.48 | 312.50 | 444.71 | 383.56 | 0.17 | -29.46 | 909.85 |
| 50 | 216.63 | 227.40 | 294.78 | 215.62 | 226.93 | 296.27 | 246.27 | 2.65 | -166.75 | 3.48 |
| 51 | 226.30 | 288.30 | 382.45 | 252.11 | 300.39 | 344.56 | 299.02 | 3.64 | -114.00 | 2248.15 |
| 52 | 260.43 | 297.98 | 273.15 | 227.23 | 282.44 | 321.89 | 277.19 | 3.83 | -135.83 | 3719.41 |
| 53 | 299.32 | 340.25 | 422.08 | 313.53 | 346.90 | 401.22 | 353.88 | 3.24 | -59.14 | 681.39 |
| 54 | 613.17 | 399.58 | 520.85 | 569.11 | 378.96 | 585.53 | 511.20 | -2.77 | 98.18 | 6549.14 |
| 55 | 347.20 | 381.38 | 475.50 | 364.75 | 389.60 | 449.73 | 401.36 | 3.01 | -11.66 | 1039.81 |
| 56 | 412.58 | 371.32 | 531.70 | 436.61 | 382.57 | 496.43 | 438.53 | 0.89 | 25.51 | 1947.97 |
| 57 | 365.70 | 291.03 | 420.12 | 366.21 | 291.27 | 419.37 | 358.95 | 0.33 | -54.07 | 0.88 |
| 58 | 655.40 | 511.05 | 705.30 | 663.61 | 514.90 | 693.24 | 623.92 | -1.65 | 210.90 | 227.68 |
| 59 | 349.88 | 365.95 | 506.45 | 382.25 | 381.10 | 458.93 | 407.43 | 2.31 | -5.59 | 3534.80 |
| 60 | 634.40 | 497.73 | 760.38 | 674.65 | 516.58 | 701.29 | 630.84 | -1.91 | 217.82 | 5466.70 |
| 61 | 135.80 | 195.23 | 234.85 | 137.12 | 195.86 | 232.90 | 188.63 | 3.92 | -224.39 | 5.92 |
| 62 | 343.92 | 343.77 | 405.47 | 337.19 | 340.62 | 415.34 | 364.38 | 2.44 | -48.64 | 152.54 |
| 63 | 434.37 | 321.43 | 461.98 | 428.53 | 318.70 | 470.56 | 405.93 | -0.61 | -7.09 | 115.06 |
| 64 | 448.20 | 284.17 | 442.78 | 435.08 | 278.02 | 462.05 | 391.72 | -1.88 | -21.30 | 581.00 |
| 65 | 690.95 | 447.12 | 878.25 | 772.74 | 485.41 | 758.17 | 672.11 | -5.38 | 259.09 | 22576.35 |
| 66 | 682.00 | 579.47 | 751.57 | 692.97 | 584.60 | 735.46 | 671.01 | -0.57 | 257.99 | 406.00 |
| 67 | 257.10 | 244.55 | 353.73 | 267.36 | 249.35 | 338.67 | 285.13 | 1.86 | -127.89 | 355.26 |
| 68 | 615.52 | 451.00 | 616.07 | 605.06 | 446.10 | 631.42 | 560.86 | -1.93 | 147.84 | 369.11 |
| 69 | 338.97 | 296.33 | 426.93 | 349.63 | 301.33 | 411.28 | 354.08 | 1.05 | -58.94 | 383.72 |
| 70 | 746.68 | 721.68 | 769.99 | 771.20 | 710.05 | 757.11 | 746.12 | -1.43 | 405.10 | 231.87 |
| 71 | 140.58 | 179.25 | 252.83 | 150.53 | 183.91 | 238.23 | 190.89 | 3.24 | -222.13 | 334.02 |
| 72 | 335.17 | 280.75 | 327.47 | 305.84 | 267.02 | 370.52 | 314.46 | 1.30 | -98.56 | 2901.50 |
| 73 | 223.95 | 231.47 | 283.23 | 215.18 | 227.36 | 296.11 | 246.22 | 2.67 | -166.80 | 259.74 |
| 74 | 462.42 | 376.87 | 498.37 | 456.40 | 374.05 | 507.19 | 445.88 | 0.13 | 32.86 | 122.00 |
| 75 | 186.85 | 163.73 | 259.67 | 188.21 | 164.37 | 257.66 | 203.42 | 1.70 | -209.60 | 6.28 |
| 76 | 229.73 | 227.42 | 323.78 | 237.45 | 231.03 | 312.45 | 260.31 | 2.17 | -152.71 | 200.95 |
| 77 | 442.02 | 299.13 | 454.25 | 433.64 | 295.21 | 466.55 | 398.47 | -1.38 | -14.55 | 236.80 |
| 78 | 262.90 | 222.30 | 332.83 | 265.44 | 223.49 | 329.11 | 272.68 | 1.22 | -140.34 | 21.70 |
| 79 | 602.30 | 340.28 | 684.45 | 640.87 | 358.34 | 627.83 | 542.34 | -5.25 | 129.32 | 5019.99 |
| 80 | 718.35 | 436.22 | 698.02 | 715.13 | 434.70 | 702.75 | 617.53 | -5.20 | 204.51 | 35.12 |
| 81 | 172.08 | 159.62 | 358.67 | 221.46 | 182.74 | 286.17 | 230.12 | 1.30 | -182.90 | 8229.33 |
| 82 | 596.47 | 402.63 | 536.48 | 563.74 | 387.31 | 584.53 | 511.86 | -2.40 | 98.84 | 3614.32 |
| 83 | 272.10 | 260.03 | 317.97 | 260.21 | 254.47 | 335.43 | 283.37 | 2.19 | -129.65 | 477.34 |
| 84 | 456.90 | 387.50 | 505.27 | 454.05 | 386.17 | 509.45 | 449.89 | 0.52 | 36.87 | 27.44 |
| 85 | 603.67 | 411.35 | 570.52 | 582.40 | 401.39 | 601.73 | 528.51 | -2.52 | 115.49 | 1525.71 |
| 86 | 357.05 | 300.75 | 480.97 | 385.25 | 313.95 | 439.57 | 379.59 | 0.43 | -33.43 | 2683.59 |
| 87 | 462.27 | 388.33 | 414.48 | 418.21 | 367.71 | 479.16 | 421.69 | 0.99 | 8.67 | 6549.09 |
| 88 | 720.08 | 485.10 | 703.70 | 712.04 | 481.33 | 715.52 | 636.29 | -3.86 | 223.27 | 218.64 |
| 89 | 428.52 | 384.17 | 501.03 | 432.70 | 386.12 | 494.90 | 437.91 | 1.09 | 24.89 | 58.96 |
| 90 | 548.25 | 372.63 | 481.57 | 510.08 | 354.76 | 537.60 | 467.48 | -1.83 | 54.46 | 4916.34 |
| 91 | 238.80 | 293.72 | 351.33 | 246.62 | 297.38 | 339.86 | 294.62 | 3.71 | -118.40 | 206.24 |
| 92 | 231.43 | 234.72 | 383.10 | 263.44 | 249.70 | 336.11 | 283.08 | 1.97 | -129.94 | 3456.98 |
| 93 | 388.73 | 310.62 | 486.20 | 408.45 | 319.85 | 457.25 | 395.18 | -0.04 | -17.84 | 1312.22 |
| 94 | 483.58 | 327.58 | 531.50 | 492.55 | 331.78 | 518.33 | 447.56 | -1.98 | 34.54 | 271.36 |
| 95 | 458.10 | 348.55 | 494.77 | 455.73 | 347.44 | 498.25 | 433.81 | -0.57 | 20.79 | 18.98 |
| 96 | 424.07 | 298.98 | 451.10 | 419.66 | 296.92 | 457.57 | 391.38 | -0.96 | -21.64 | 65.55 |
| 97 | 336.68 | 250.53 | 332.80 | 313.42 | 239.64 | 366.95 | 306.67 | 0.36 | -106.35 | 1825.66 |
| 98 | 476.37 | 365.78 | 507.40 | 471.69 | 363.59 | 514.27 | 449.85 | -0.56 | 36.83 | 73.88 |
| 99 | 507.63 | 401.63 | 543.48 | 504.41 | 400.13 | 548.21 | 484.25 | -0.46 | 71.23 | 34.97 |
| 100 | 605.80 | 485.80 | 649.55 | 607.96 | 486.81 | 646.38 | 580.38 | -0.91 | 167.36 | 15.75 |
| 101 | 331.82 | 200.40 | 304.20 | 304.51 | 187.62 | 344.29 | 278.81 | -0.80 | -134.21 | 2515.92 |
| 102 | 869.03 | 817.03 | 874.00 | 879.01 | 816.01 | 865.03 | 853.35 | -1.57 | 88.00 | 257.65 |
| 103 | 469.33 | 343.57 | 508.52 | 470.31 | 344.02 | 507.09 | 440.47 | -1.05 | 27.45 | 3.20 |
| 104 | 680.92 | 488.60 | 699.40 | 682.12 | 489.16 | 697.64 | 622.97 | -2.84 | 209.95 | 4.86 |
| 105 | 593.07 | 424.10 | 601.67 | 586.73 | 421.13 | 610.97 | 539.61 | -2.11 | 126.59 | 135.59 |
| 106 | 547.60 | 335.58 | 649.15 | 587.67 | 354.34 | 590.32 | 510.78 | -3.93 | 97.76 | 5418.96 |
| 107 | 458.97 | 418.32 | 557.50 | 473.95 | 425.33 | 535.50 | 478.26 | 1.04 | 65.24 | 757.81 |
| 108 | 387.73 | 296.22 | 539.27 | 432.83 | 317.33 | 473.05 | 407.74 | -0.76 | -5.28 | 6864.14 |
| 109 | 599.78 | 356.45 | 573.48 | 588.58 | 351.20 | 589.93 | 509.91 | -4.04 | 96.89 | 423.69 |
| 110 | 465.38 | 379.37 | 511.80 | 463.99 | 378.71 | 513.85 | 452.18 | 0.05 | 39.16 | 6.56 |
| 111 | 653.83 | 404.10 | 680.23 | 666.44 | 410.00 | 661.72 | 579.39 | -4.55 | 166.37 | 536.64 |
| 112 | 402.18 | 357.58 | 398.62 | 373.30 | 344.06 | 441.03 | 386.13 | 1.56 | -26.89 | 2815.80 |
| 113 | 564.87 | 394.45 | 605.67 | 572.74 | 398.13 | 594.11 | 521.66 | -2.35 | 108.64 | 209.08 |
| 114 | 605.00 | 491.10 | 676.12 | 618.22 | 497.29 | 656.71 | 590.74 | -0.91 | 177.72 | 589.77 |
| 115 | 418.90 | 343.40 | 398.18 | 386.84 | 328.39 | 445.25 | 386.83 | 0.77 | -26.19 | 3468.66 |
| 116 | 637.02 | 350.50 | 531.63 | 597.40 | 331.95 | 589.80 | 506.38 | -4.79 | 93.36 | 5297.22 |
| 117 | 337.05 | 326.15 | 445.50 | 352.22 | 333.25 | 423.22 | 369.57 | 1.83 | -43.45 | 776.83 |
| 118 | 936.23 | 653.73 | 908.83 | 929.99 | 650.81 | 918.01 | 832.93 | -1.44 | 419.91 | 131.81 |
| 119 | 649.15 | 603.80 | 772.42 | 675.55 | 616.16 | 733.66 | 675.12 | 0.75 | 262.10 | 2351.59 |
| 120 | 859.57 | 651.42 | 810.80 | 833.71 | 639.31 | 848.77 | 773.93 | -2.88 | 360.91 | 2256.82 |
